# Shape‐Determined Kinetic Pathways in 2D Solid–Solid Phase Transitions

**DOI:** 10.1002/advs.202517016

**Published:** 2025-11-03

**Authors:** Ruijian Zhu, Yi Peng, Yanting Wang

**Affiliations:** ^1^ Institute of Theoretical Physics Chinese Academy of Sciences Beijing 100190 China; ^2^ Beijing National Laboratory for Condensed Matter Physics Institute of Physics Chinese Academy of Sciences Beijing 100190 China; ^3^ School of Physical Sciences University of Chinese Academy of Sciences 19A Yuquan Road Beijing 100049 China

**Keywords:** ball‐stick polygon, kinetics, molecular dynamics simulation, soft matter, solid–solid phase transition

## Abstract

Solid–solid phase transitions are ubiquitous in nature, but the kinetic pathway of anisotropic particle systems remains elusive, where the coupling between translational and rotational motions plays a critical role in various kinetic processes. Here this problem is investigated by molecular dynamics simulation for 2D ball‐stick polygon systems, where pentagon, hexagon, and octagon systems all undergo an isostructural solid–solid phase transition. During heating, the translational motion exhibits merely a homogeneous expansion, whereas the time evolution of body‐orientation is shape‐determined. The local defects of body‐orientation self‐organize into a vague stripe for pentagon, a random pattern for hexagon, while a distinct stripe for octagon. The underlying kinetic pathway of octagon adheres to the quasi‐equilibrium assumption, whereas those of hexagon and pentagon are predominantly governed by translational motion and rotational motion, respectively. This diversity is originated from different kinetic coupling modes determined by the anisotropy of molecules, and can affect the phase transition rates. The reverse process in terms of cooling follows the same mechanism, with more diverse kinetic pathways attributed to the possible kinetic traps. These findings promote theoretical understanding of microscopic kinetics of solid–solid phase transitions as well as provide direct guidance for the rational design of materials utilizing desired kinetic features.

## Graphical abstract

By simulating 2D ball‐stick pentagon, hexagon, and octagon systems, the kinetics of solid–solid phase transition is investigated, which is characterized by the loss of body‐orientation order. During which, the local defects of body‐orientation self‐organize into three different patterns, originated from the shape‐determined kinetic pathways, which may satisfy the quasi‐equilibrium assumption or be dominated by either the translational motion or the rotational motion.

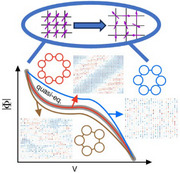

## 1 Introduction

Solid–solid (s–s) phase transition is one of the most ubiquitous types of phase transitions in natural and man‐made materials, including alloys,^[^ ^1^ ^]^ minerals,^[^ ^2^ ^]^ ionic liquids,^[^ ^3^ ^]^ ice^[^ ^4^, ^5^ ^]^ and biological processes.^[^ ^6^ ^]^ S–s transitions also hold considerable technological importance in earth science,^[^ ^7^, ^8^ ^]^ diamond production,^[^ ^9^ ^]^ memory alloys,^[^ ^10^, ^11^, ^12^ ^]^ reconfigurable optical devices^[^ ^13^ ^]^ and reprogramming self‐assembly.^[^ ^14^, ^15^, ^16^, ^17^ ^]^ Specifically, 2D molecular crystals stabilized by non‐covalent interaction have become a hot topic in materials science and chemical engineering,^[^ ^18^, ^19^, ^20^ ^]^ where the s–s transition has already been observed and is suggested to be useful for molecular electronic devices.^[^ ^21^ ^]^ Beyond thermodynamic aspect, it has been shown that different kinetic pathways can influence both phase transition rates^[^ ^22^, ^23^, ^24^ ^]^ and final products^[^ ^23^, ^25^ ^]^ in the s–s transitions. The kinetic pathway follows the free energy landscape if the quasi‐equilibrium assumption is satisfied, but can also deviate when kinetic details are non‐negligible.^[^ ^26^ ^]^

In order to investigate the microscopic kinetics of s–s transitions, spherical colloids, benefiting from the single‐particle‐resolution and the controllability of interactions and sizes,^[^ ^27^ ^]^ serve as outstanding model systems. To date, extensive studies have revealed various kinetic pathways modulated by external stress,^[^ ^24^, ^28^, ^29^ ^]^ electric field,^[^ ^23^ ^]^ grain boundaries,^[^ ^24^, ^30^, ^31^ ^]^ and internal softness.^[^ ^30^ ^]^ However, spherical colloids are not suitable for studying kinetic pathways of s–s transitions in anisotropic systems, where monomer shape critically impacts the transition pathway via kinetic coupling between translational and rotational motions, as demonstrated in crystallization,^[^ ^32^, ^33^, ^34^, ^35^ ^]^ vitrification,^[^ ^36^ ^]^ and the formation of polycrystals.^[^ ^37^ ^]^ Although s–s transitions have also been observed in a variety of 3D^[^ ^22^, ^38^, ^39^, ^40^ ^]^ and 2D^[^ ^35^, ^41^, ^42^, ^43^, ^44^, ^45^, ^46^, ^47^, ^48^ ^]^ systems composed of anisotropic particles through molecular simulations or colloidal experiments, prior works predominantly focus on either thermodynamics or kinetics of translational motion only.

**Figure 1:**
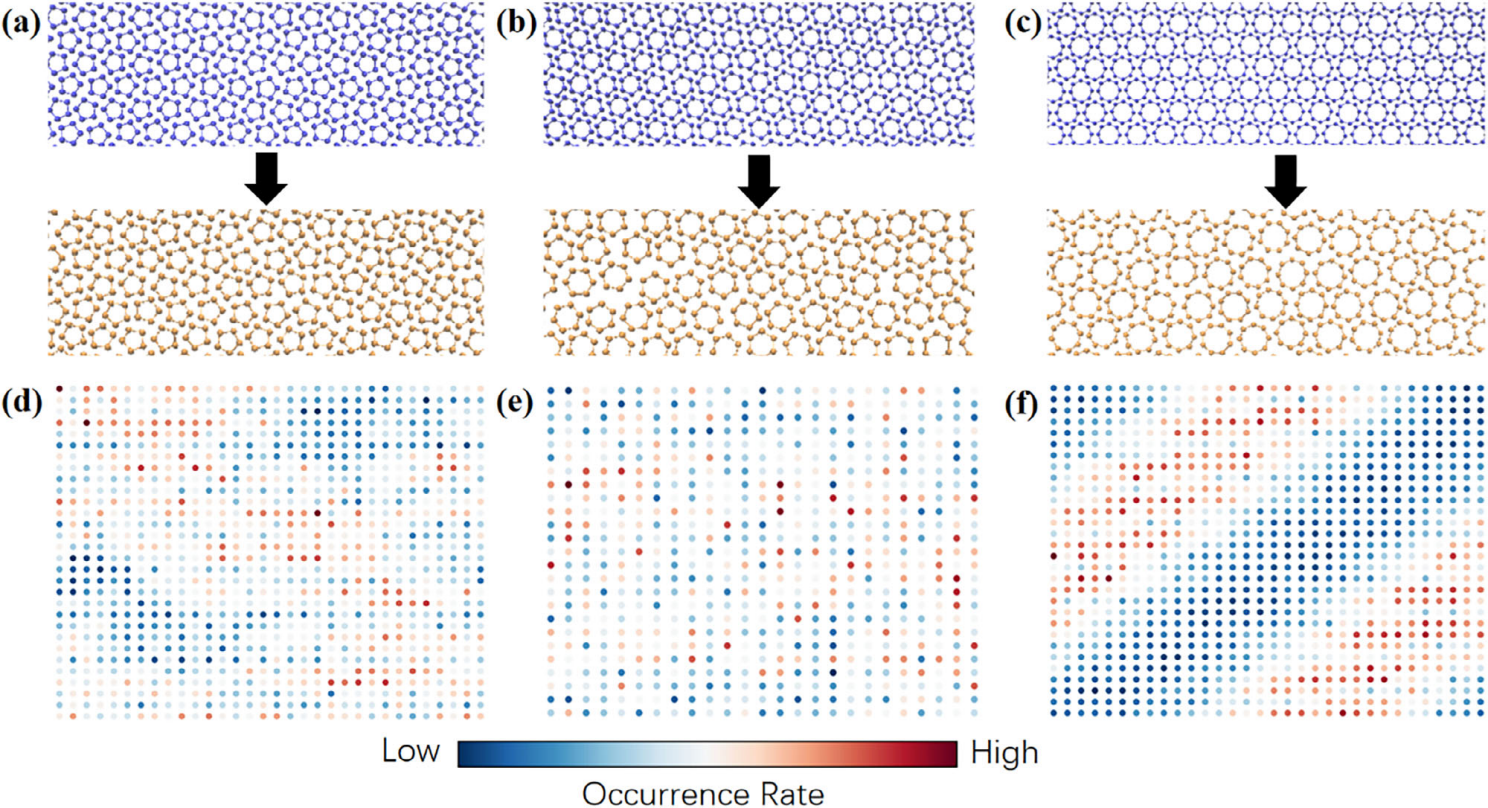
Crystalline morphologies and rotational motions of the s–s transitions in three ball‐stick polygon systems. a) Crystal states of pentagon. b) Crystal states of hexagon. c) Crystal states of octagon. a–c) The upper panels are the snapshots of ground‐state morphologies with monomers colored in blue, sharing an ordered body‐orientation, and the lower panels are the snapshots of rotator crystal state with monomers colored in orange, exhibiting random body‐orientations. d–f) The spatial distributions of local defects in the body‐orientation fields with warmer colors indicating a higher defect occurrence rate. The points with high occurrence rates form a vague striped region along with some randomly distributed points in d) the pentagon system, exhibit a highly random pattern in e) the hexagon system, and demonstrate a distinct striped region across the box in f) the octagon system. The results showed here are all obtained at the lowest investigated pressure of the corresponding polygon (*P* = 0 for pentagon and octagon while *P* = 1.5 for hexagon).

Our recent theoretical study^[^ ^48^ ^]^ on 2D ball‐stick polygon systems, composed of one L‐J ball on each vertex and valence bonds between adjacent balls, has revealed complex phase behaviors despite the simplicity of the model. Specifically, the ball‐stick pentagon, hexagon, and octagon all exhibit s–s transitions from the close‐packing phase to the rotator crystal phase during heating at an appropriate pressure, whose structures are shown in **Figure** **1**a–c. These s–s transitions are isostructural,^[^ ^49^, ^50^ ^]^ as observed in some colloidal systems^[^ ^51^, ^52^ ^]^ and colloid–polymer mixtures,^[^ ^53^ ^]^ where the lattice homogeneously expands at the phase transition point drastically with its symmetry group retained. Given that their translational motions are well‐understood, these ball‐stick polygons are ideal for exploring how the coupling between translational and rotational motions affects the kinetic pathways of s–s transitions in anisotropic particle systems.

In this work, we investigate the kinetics of the s–s phase transitions of ball‐stick pentagon, hexagon, and octagon systems by molecular dynamics (MD) simulation. During heating, the amorphization of the well‐ordered body‐orientations in the parent phase occurs concurrently with the homogeneous expansion. The rotational motion is quantified by the local defects of the body‐orientation, which arrange into three qualitatively different patterns: The defects for pentagon form a vague stripe, the ones for hexagon create a random pattern, whereas the ones for octagon produce a distinct stripe. Our mechanistic analysis reveals shape‐determined kinetic pathways originated from different kinetic coupling modes: pentagon has a kinetic pathway dominated by rotational motion, hexagon is dominated by translational motion, and octagon satisfies the quasi‐equilibrium assumption where the rotational and translational motions are synchronous. Different kinetic pathways can directly influence the phase transition rate. The reverse process follows the same mechanism with more diverse kinetic pathways due to kinetic traps. By examining the local structures of the crystalline states for different polygons, we conclude that a stronger rotational constraint promotes a kinetic pathway preferring translational motion and favoring rotational motion in the reverse process, and vice versa.

## 2 Results

### 2.1 Macroscopic Features of S–S Transitions

As shown in Figure 1a–c, the ground state of the ball‐stick pentagon system adopts a striped phase,^[^ ^48^ ^]^ where monomers are aligned alternatively along two parallel body‐orientations, and their center‐of‐masses (COMs) form a triangular‐lattice structure; for both hexagon and octagon, the ground states are triangular‐lattice with uniform body‐orientation. The balls form a structure closed to distorted honeycomb lattice in the hexagon system, but no clear lattice structures formed in pentagon and octagon systems. Throughout this paper, the term monomer refers to one ball‐stick polygon and only the lattice structures of COMs are concerned. Under an appropriate temperature, the lattice undergoes a sudden expansion, leading to a sharp increase in potential energy and a concurrent decrease in density, which are typical features for a discontinuous phase transition. This rise in potential energy is compensated by the larger entropy gained from the loss of body‐orientational order, resulting in a rotator crystal phase that preserves only translational order, evidenced by a more uniform and broader distribution of body‐orientation angle.^[^ ^48^ ^]^ Both the parent and product phases in these three systems can be characterized by the bond‐orientational order parameter Ψ_6_,^[^ ^48^ ^]^ manifesting a six‐fold symmetry of the lattices. The same symmetry between parent and product lattices confirms the isostructural nature of these s–s transitions.^[^ ^49^, ^50^ ^]^ Despite the similar thermodynamic behavior, it would be interesting to know whether the kinetic pathways of the s–s transitions for different polygons exhibit shape‐dependent features.

Before exploring the microscopic details of kinetics, let us first take a look at the general features of translational and rotational motions. Starting from the ground‐state crystalline morphology equilibrated at a temperature slightly below the phase transition point, the s–s transition is triggered by heating the system across the phase transition point. During the transition, the lattice undergoes a homogeneous spatial expansion, quantified by the time evolution of Voronoi cell areas associated with the lattice points, as shown in Figure S1a–c (Supporting Information). The area distribution retains a unimodal shape throughout the transition, with its peak continuously shifting toward the direction corresponding to a larger area. This behavior is observed in all the three polygons, consistent with the previous theoretical prediction.^[^ ^49^, ^50^ ^]^ The inter‐monomer distances of the expanding lattice increase, leading to a greater free volume for the particle to rotate. Consequently, once the density of the system starts decreasing, the body‐orientational parameter defined as ϕ_*i*_ = exp ( inθ_*i*_) also begins to decrease, as can be seen in Figure S2 (Supporting Information). Here θ_*i*_ is the angle between an arbitrary vertex of polygon *i* and the *x*‐axis, and *n* takes the *n*‐fold symmetry of a polygon into account. For hexagon and octagon, *n* is just the edge number, whereas for pentagon, *n* is set to 10 because there are two alternative body‐orientations in the ground state. When the body‐orientation of the monomers becomes disordered, the lattice takes several more steps to relax to its final structure, forming a rotator crystal.

### 2.2 Defects in the Body‐Orientation Field

More detailed features of the rotational motion are obtained by examining the body‐orientation field, defined as a collection of unit vectors located at the monomer COMs, whose directions represent the arguments of the body‐orientational order parameters of the monomers with respect to the *x*‐axis. All vectors are roughly aligned along the same direction in the parent phase, and become disordered in the product phase. To quantify this amorphization process, we track the production and annihilation of local defects (defined in the Experimental section), which remark the positions where body‐orientations change drastically.

For the pentagon system, some defects already present in the parent phase, as shown in Figure S3a in Supporting Information. As the transition proceeds, the number of defects increases significantly, which initially form a striped region across the simulation box and eventually become uniformly distributed, as shown in Figure S3b–f (Supporting Information). This feature is quantified by the spatial distribution of local defects (detailed in the Experimental section). By averaging over all the snapshots sampled during the transition, a vague striped region exhibits along the crystal axis direction across the box, as shown in Figure 1d, alongside isolated defects wherever easy to create without significant expansion.

For the hexagon system, very few defects are present in the parent phase, as shown in Figure S4a (Supporting Information). As the transition progresses, defects begin to appear randomly in the box, as shown in Figure S4b–d (Supporting Information). Unlike the pentagon system, no regular patterns emerge during the entire transition process. The spatial distribution of defects shown in Figure 1e is totally random, coinciding with the snapshots shown in Figure S4 (Supporting Information).

During the transition process, the octagon system initially behaves similarly to the hexagon system and later resembles the behavior of the pentagon system. A very small number of randomly distributed defects initially appear and subsequently disappear due to thermal fluctuations before the formation of a stable narrow stripe across the box. The striped region then extends along its normal direction via a rate‐limited process, until finally uniformly covers the whole simulation box. The typical snapshots demonstrating the above process are presented in Figure S5 (Supporting Information). The distinct stripe across the box for the spatial distribution of local defects appeared in Figure 1f supports the above physical picture.

### 2.3 Diversity of Kinetic Pathways

The above three observed patterns imply a shape‐determined nature of the kinetic pathway. To reveal the underlying mechanism in terms of kinetic coupling modes of translational and rotational motions, it is instructive to fix the translational motion while allow the monomers to rotate freely in MD simulation (hereafter referred as “fixed simulation”). For the fixed simulations, we randomly select several conformations along the transition process in the original trajectory to serve as initial configurations with the COM position of each monomer being fixed, and then perform MD simulations in the NVT ensemble (more details can be found in the Experimental section and Supporting Information). Since the lattice homogeneously expands, each snapshot can represent the COM positions at a given volume. Therefore, this constrained system spontaneously relaxes to the equilibrium state given by the restricted free energy *F*(*V*, Φ), where the ensemble average of body‐orientational order parameter Φ directly corresponds to the minimum satisfying ${\left(\frac{\partial F(V,\Phi )}{\partial \Phi }\;\right)}_{V}=0$. Below we present the ensemble‐averaged results of Φ and compare them with the values obtained from the unfixed simulation, i.e., the original MD simulation, with the same volume. More information regarding the time evolution of the body‐orientation and the selection of initial configurations for the fixed simulations can be found in Supporting Information.

**Figure 2:**
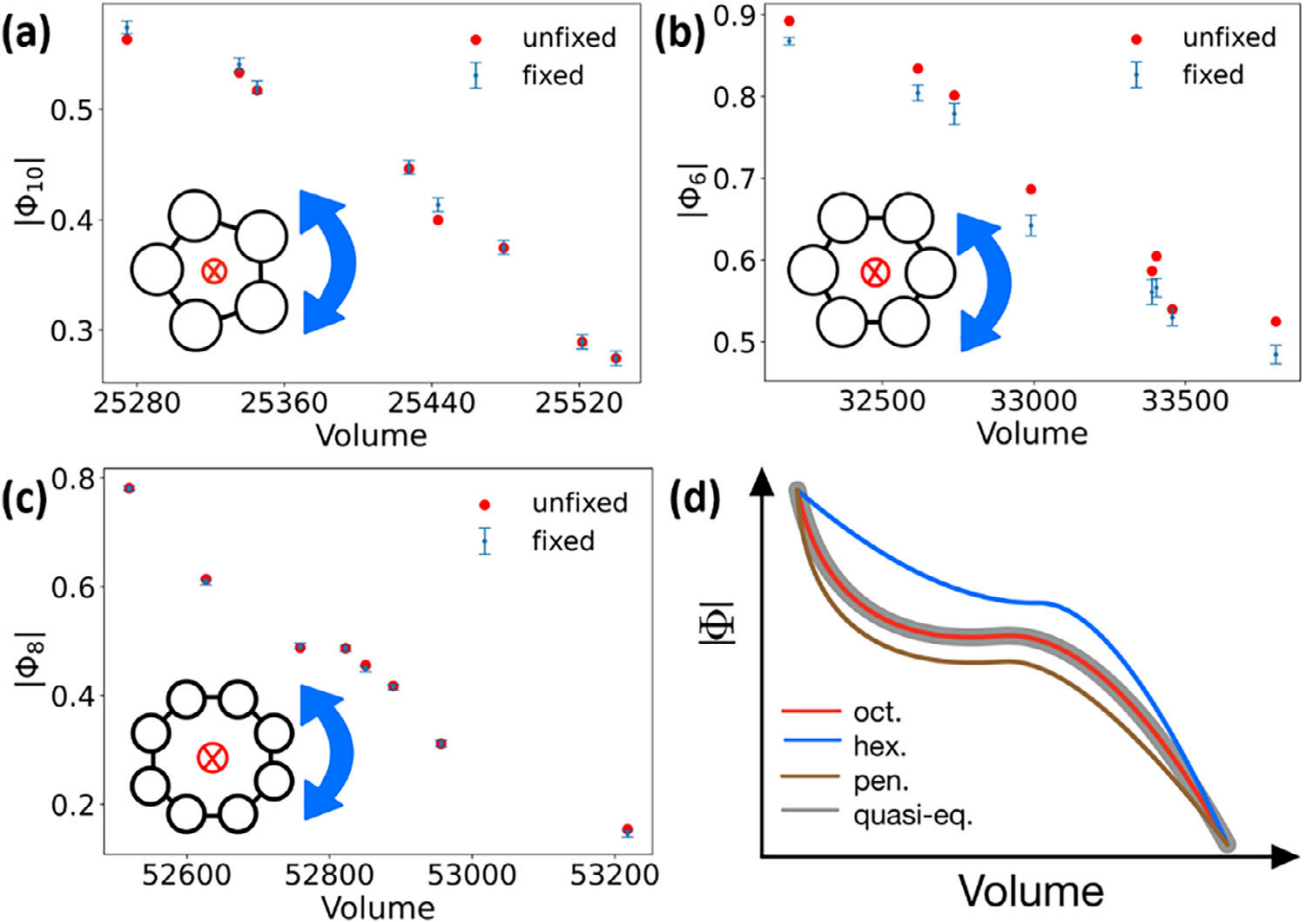
The results from the fixed simulations. a) Pentagon: The equilibrium values from the fixed simulations are slightly larger than or equal to the values from the unfixed MD simulation. b) Hexagon: The equilibrium values from the fixed simulations are consistently and significantly smaller than the values from the unfixed MD simulation. c) Octagon: The equilibrium values from the fixed simulations are always equal to the values from the unfixed MD simulation. The inset of each subfigure illustrates that, in the fixed simulations, each monomer has its COM fixed but is totally free to rotate. a–c) Each data point labeled “fixed” comes from an individual fixed simulation, and each point labeled “unfixed” comes from a single trajectory of a regular MD simulation by heating up the parent phase. d) Schematic illustration of the real kinetic pathways and the pathway satisfying the quasi‐equilibrium assumption.

The fixed simulations have revealed three qualitatively different kinetic pathways for the polygon systems: 1) For pentagon, as shown in **Figure** **2**a, the fixed simulations give slightly larger or equal equilibrium values of Φ than the unfixed simulation, indicating that the rotational motion is marginally faster than the translational motion, i.e., the kinetic process is led by rotation; 2) By contrast, for hexagon, the fixed simulation always produce a smaller equilibrium value of Φ than the unfixed simulation, as shown in Figure 2b, suggesting that the translational motion is faster and the transition process is dominated by expansion; 3) The case for octagon shown in Figure 2c falls in between, where the equilibrium values of Φ from the fixed simulations are consistently identical with the unfixed simulations, indicating that the rotational and translational motions are synchronous in the transition process.

The kinetic pathways of s–s transitions are widely regarded as following the thermodynamic free energy landscape,^[^ ^22^, ^54^ ^]^ which however, makes sense only if the quasi‐equilibrium assumption is satisfied.^[^ ^26^ ^]^ This assumption requires that the kinetic pathway follows exactly the one connecting the minima of the constrained free energy values at different volumes, i.e., the pathway connecting the points satisfying $\frac{\partial F(V,\Phi )}{\partial \Phi }=0$ for all possible volumes *V*. As schematically illustrated in Figure 2d, the kinetic pathway of pentagon is featured by a faster evolution of body‐orientation than in the quasi‐equilibrium pathway given by the thermodynamic free energy landscape, the one of hexagon suggests a faster evolution of volume, while only the one of octagon follows the quasi‐equilibrium pathway. The violation of the quasi‐equilibrium assumption suggests that the details of kinetics are non‐negligible for the transition process.^[^ ^26^ ^]^ Furthermore, it should be noted that the kinetic pathway dominated by rotation is close to the quasi‐equilibrium one, because the rotational motion is significantly retarded by the repulsion of neighboring monomers; on the other hand, expansion faces no explicit obstacles, allowing the pathway dominated by the translational motion to be far from the quasi‐equilibrium one.

The identified kinetic pathways can effectively explain the patterns of defects shown in Figure 1d–f. In the hexagon system, where the expansion dominates the kinetic process, the relatively large distance between adjacent monomers allows the rotational motion to occur independently, resulting in a random pattern of local defects. By contrast, as for the case of pentagon and octagon, the neighboring monomers hinder the rotational motion due to the small interparticle distance. Consequently, when one particle changes its body‐orientation due to thermal fluctuation, it is more likely to induce a rotation for its neighboring monomers rather than those located far away. This is analogous to the fact that the second low‐energy excitation state of 2D Ising model is flipping two adjacent spins instead of two separated ones.^[^ ^55^ ^]^ The defects then propagate along one of the crystalline axes, forming a narrow striped region, which resembles a typical configuration of phase coexistence in 2D system with periodic boundary condition.^[^ ^56^ ^]^ The difference between pentagon and octagon is that the rotational motion is relatively easy in the pentagon system, indicated by the existence of a small number of defects in the parent phase, which results in some isolated defects besides the main striped region.

Although the above results are all obtained at the lowest pressures (*P* = 0 for both pentagon and octagon, while *P* = 1.5 for hexagon, more discussions can be found in the Experimental section) for the s–s transitions, more simulations at various pressures have shown that both the kinetic pathways and the patterns of local defects in the body‐orientation field are robust with respect to the pressure, indicating that they only rely on intrinsic properties of monomers. The simulation results at other pressures can be found in Supporting Information.

### 2.4 Phase Transition Rate

**Figure 3:**
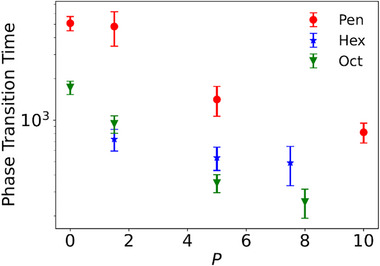
Phase transition times for various polygons. Each data point is averaged over ten independent simulation trajectories. Five of the ten trajectories are simulated at *T*_m_, while the other five are simulated at *T*_m_ + 0.01. The phase transition time is expressed in the unit of $\tau \equiv \sqrt{m\sigma^{2}/\varepsilon }$. The *y*‐axis adopts a logarithmic scale. The error‐bars represent the standard deviation, resulting from the randomness among different simulation trajectories.

The diversity of kinetic pathways directly affects the phase transition rate, which is quantified in this work by the simulation time required to finish the phase transition (detailed in the Experimental section). The starting point of the transition is selected as the moment when the potential energy of the system significantly deviates from the average value in the parent phase without reverting, and the endpoint is chosen as the time when the potential energy of the system reaches the average value in the product phase without obvious decrease thereafter. Although the choices of the starting and ending points are not very rigorous, they should not affect the qualitative conclusions we draw. As depicted in **Figure** **3**, hexagon exhibits a relatively stable phase transition rate across various pressures, whereas both pentagon and octagon show a significantly accelerated transition rate as the pressure increases. Combining with the thermodynamic data shown in Table S1 (Supporting Information), it can be inferred that the energy barrier is lower at a higher pressure due to reduced structural difference between the parent and product phases. For pentagon and octagon, where the rotational motion is pivotal in the transition process, the faster transition rate benefits from the lower energy barrier. This is also visible from the body‐orientation field shown in Figures S6, S8, S9, and S11 (Supporting Information), where the time required to form a narrow stripe is nearly constant at various pressures, but the growth of the stripe along its normal direction is faster at higher pressures. In the case of hexagon, the kinetic process is dominated by expansion, which has little to do with the energy barrier retarding rotational motion. Since the increases in phase‐transition temperature and pressure balance each other, the hexagon system has a relatively stable speed for the translational motion, and thus the overall transition rate.

### 2.5 Reverse Process

**Figure 4:**
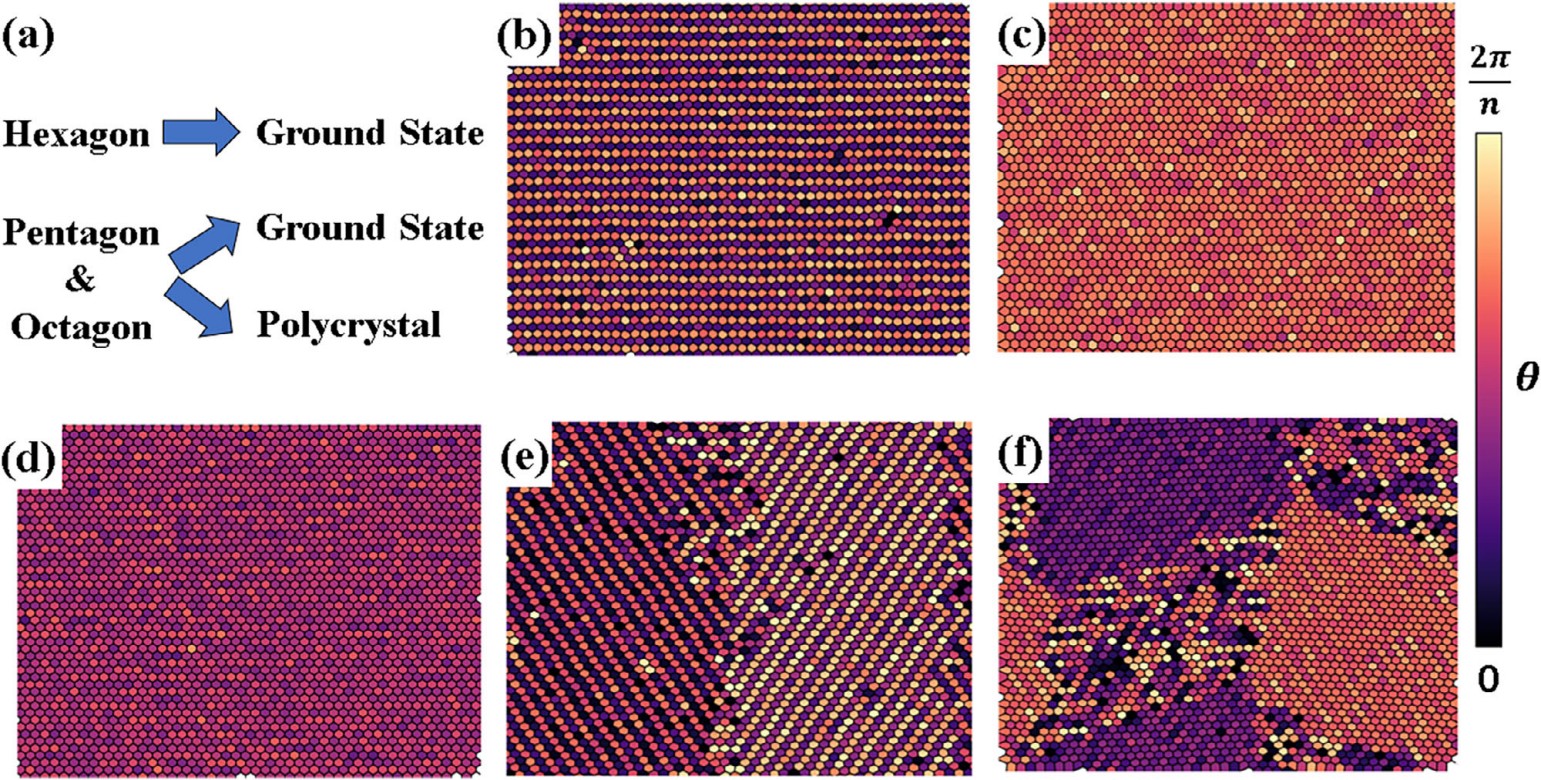
Final structures of the reverse processes. a) Summary of the result that hexagon always ends up with the ground‐state morphology (perfect lattice) while pentagon and octagon can reach either the perfect lattice or a polycrystal. b) Perfect lattice of pentagon. c) Perfect lattice of hexagon. d) Perfect lattice of octagon. e) Polycrystalline morphology of pentagon. f) Polycrystalline morphology of octagon. The polycrystals basically consist of two pieces of perfect crystals with different orientations. b–f) Each Voronoi cell associated with the lattice points is colored according to the body‐orientation angle θ of corresponding monomer, ranging from 0 to 2π/*n*.

The reverse process is studied by cooling down the rotator crystal state, during which the area of each Voronoi cell associated with the lattice points still retains a unimodal distribution, in agreement with the theoretical picture of homogeneous contraction,^[^ ^49^, ^50^ ^]^ as shown in Figure S1d–f (Supporting Information). The overall results for the three polygon systems are summarized in **Figure** **4**a. It is quite interesting that the reverse process for pentagon and octagon may approach either the corresponding ground state, as shown in Figure 4b,d, or a metastable polycrystalline state formed by two large crystal fragments with different crystalline axes due to the kinetic trap. Both fragments adopt the ground‐state crystalline structure, as depicted in Figure 4e for pentagon and f) for octagon. Conversely, all six of our reverse simulations for hexagon consistently result in the ground state shown in Figure 4c. Since it is possible to reach a monocrystalline structure via cooling, it offers a route for recovering monocrystalline 2D materials from polycrystals by first heating it across the s–s transition temperature and then cooling it down.

To understand the effect of kinetic coupling in the reverse process, we also perform the fixed simulations for each polygon. The results suggest that the same polygon can have different kinetic pathways, as demonstrated in Figure S15 (Supporting Information). Despite the enhanced randomness compared to the heating process, the coupling mode has a strong correlation with the final product: When the kinetic process is dominated by contraction, the final product is always polycrystal; when it is dominated by rotation or satisfies the quasi‐equilibrium assumption, the product forms a perfect crystal. In the scenarios where the rotational motion is faster, the monomers can effectively adjust their orientations. However, when translational motion prevails, the system is compressed into a high‐density state, resulting in a kinetic trap devoid of global alignment of the monomers.

### 2.6 Connection between Kinetic Behaviors and Monomer Properties

**Figure 5:**
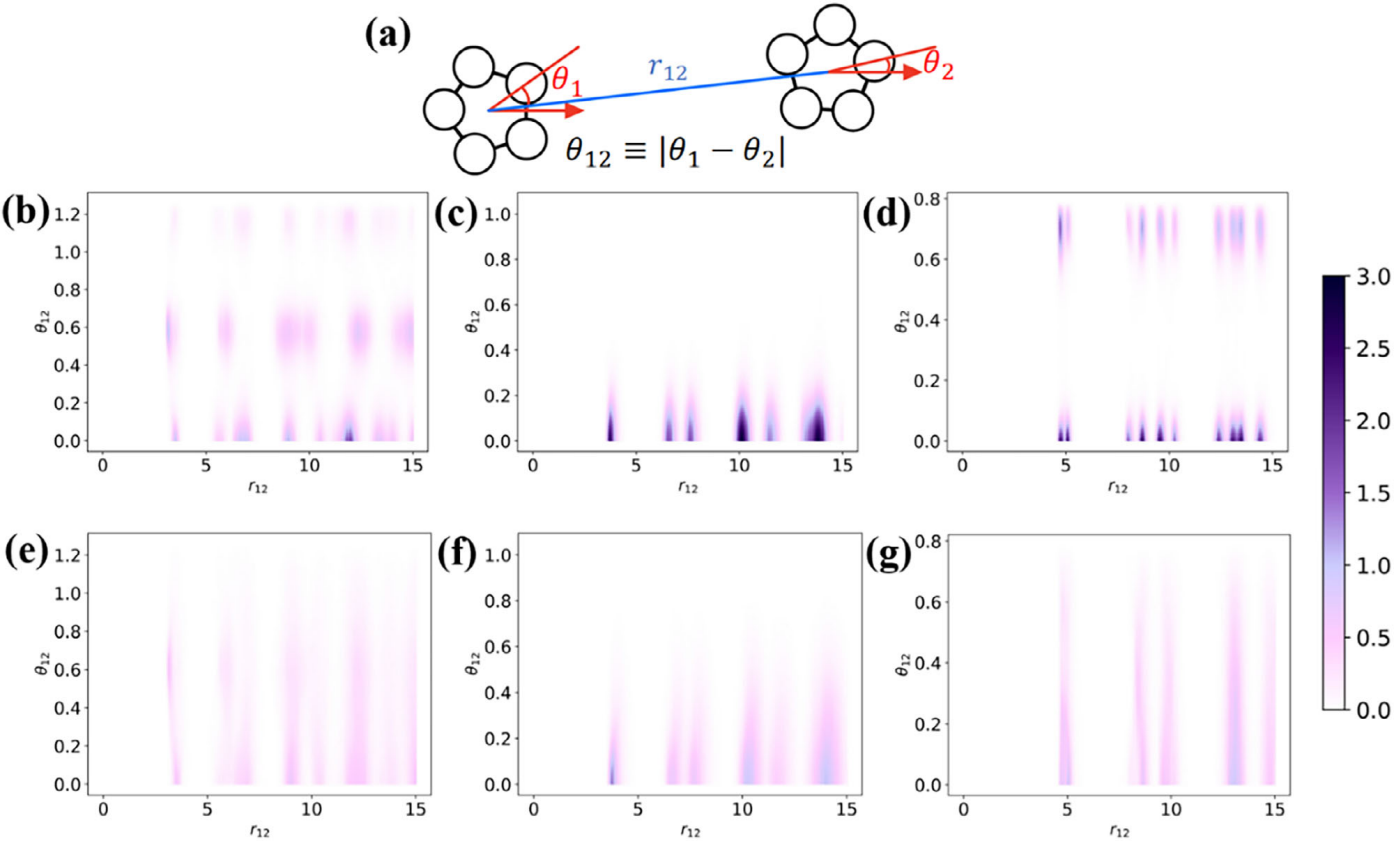
Probability density maps of relative angle (θ_12_) and relative distance (*r*_12_) between two monomers. a) Schematic illustration of the definitions of θ_12_ and *r*_12_. b–d) are calculated in the close‐packing phase and (e–g) are calculated in the rotator crystal phase. b,e): pentagon. c,f): hexagon. d,g): octagon. Since the range of θ_12_ is different for different polygons, the probability density is normalized by the case of the uniform distribution.

In order to guide material design for targeted kinetic properties in s–s transitions, it is essential to relate the observed kinetic behaviors to molecular properties in terms of shape and interaction. These molecular properties govern the local structure in the close‐packing phase, which in turn influences the phase transition kinetics. We quantify this local structure through statistical analyses of the relative distance *r*_12_ and the relative body‐orientation angle θ_12_, which is defined as the absolute value of the difference in body‐orientation θ between two monomers, as illustrated in **Figure** **5**a. The absolute value guarantees a larger θ_12_ value simply corresponds to a larger deviation of the relative body‐orientation between neighboring monomers. The *n*‐fold symmetry is taking into account by limiting the value of θ in the range of 0 to 2π/*n*, where *n* is the edge number of each polygon. As depicted in Figure 5b–d, the ground state of pentagon exhibits a highly dispersive distribution of θ_12_, hexagon displays several distinct peaks with small dispersion, while octagon also suggests a centralized distribution, but approximately half of the neighbors of a monomer significantly deviate from its orientation. Because a stronger rotational constraint implies that the monomer in the crystalline state is difficult to change its body‐orientation relative to its neighbors, these results indicate that pentagon has the weakest rotational constraint on neighboring monomers, octagon has an intermediate constraint, and hexagon has the strongest one.

A qualitative understanding on the rotational constraint can be obtained from the consideration on simply the shape of the monomer. Polygon with less edges exhibits a stronger orientational entropy,^[^ ^57^ ^]^ so it is expected that hexagon has a stronger rotational constraint than octagon. The pentagon, though with less edges than hexagon and octagon, has interlaced body‐orientations of monomers in the striped phase, which significantly weakens the constraint on adjacent monomers with opposite orientations.

Because the interaction strength generally decays with the distance between monomers, when a strong rotational constraint is present, the system tends to expand before rotation to effectively attenuate the constraint. Conversely, with a weak constraint, the polygon can rotate without significant expansion.

Performing the same statistical analyses in the rotator crystal phase can also identify a direct connection between rotational constraints and kinetic behaviors. As shown in Figure 5e–g, pentagon and octagon almost have no constraints on the relative angle of neighboring monomers, so the adjustment of the body‐orientation cannot take place before contraction. As a result, the monomers may not align globally before contracted into a dense state. The hexagon, however, retains a moderate constraint on the relative angle, ensuring adequate modification of body‐orientation before significant contraction.

## 3 Conclusions

In this work, we have revealed diverse kinetic pathways of s–s phase transitions in the 2D ball‐stick polygons (pentagon, hexagon, and octagon) by MD simulation. During the transition from close‐packing state to rotator crystal state, we have identified three different patterns for the time evolution of the body‐orientation field, originated from the different kinetic‐coupling modes between translational and rotational motions. The underlying kinetic pathway is shape‐determined, either being dominated by one of the motions or satisfying the quasi‐equilibrium assumption. One of the direct consequences is that the phase‐transition rate is roughly constant at various pressures if translational motion is dominant, and otherwise it becomes faster at higher pressures. In the reverse process of cooling down the rotator crystal phase, the products of pentagon and octagon are either perfect crystals or polycrystals, whereas the product of hexagon is consistently the perfect crystalline structure. The final structure is strongly correlated with the kinetic pathway of this transition process. All the kinetic behaviors can be linked to the monomer properties in terms of the rotational constraints.

Despite the extensive studies on translational motion, the literature is less focused on the kinetic coupling between translational and rotational motions in s–s transitions, which is prevalent in almost all anisotropic particle systems and known to be crucial for various kinetic processes. Consequently, there is a gap between theoretical studies and novel s–s transitions in real material systems. Benefiting from the simplicity of the translational motion of the isostructural transitions, ball‐stick polygon systems, exhibiting transitions from close‐packing state to rotator crystal state, provide us an ideal platform for examining the effect of the kinetic coupling. The diversity of kinetic pathways, especially the ones violating the quasi‐equilibrium assumption, emphasizes the importance of considering detailed kinetics, rather than merely concentrating on the thermodynamic free energy landscape. It has been shown that monomers ranging from atomic scale to macromolecules have similar kinetic and thermodynamic behaviors only if they share the same symmetry of shape and interaction.^[^ ^58^ ^]^ Our results, which have been directly connected to monomer properties, are expected to be valid in a variety of realistic systems composed of 2D polygonal components, e.g., benzene, coronene, circumcoronenes,^[^ ^59^, ^60^ ^]^ kekulene,^[^ ^61^ ^]^ and patchy colloids with designed symmetry.^[^ ^62^ ^]^ Therefore, this work not only enhances theoretical understanding of the microscopic kinetics of the s–s transitions, but also provides guidance for the rational design of molecular devices with desired kinetic properties.

There are still some limitations in this work. For instance, in chemical synthesis or cell tissue,^[^ ^63^, ^64^ ^]^ system usually shows polydisperse shape, and therefore a polygon mixture^[^ ^65^ ^]^ should be considered. Moreover, the mechanism we propose here may be extended to s–s transitions with more complicated translational motion, e.g., the four‐patch colloid with a set of inter‐patch angles of (90°, 90°, 90°, 90°) exhibiting a transition from a square lattice to a triangular lattice^[^ ^66^ ^]^ can be a good candidate. Lastly, it would be interesting to consider a quasi‐2D system with large fluctuations on the *z*‐direction. These problems can be investigated in the future to deepen our understanding on the effect of kinetic coupling and advance the applications in more realistic cases.

## 4 Experimental Section

### Preparation of Initial Configurations

Each ball‐stick *n*‐gon is composed of *n* L‐J balls and *n* covalent bonds connecting adjacent balls. The covalent bond is represented by a harmonic potential with a very large strength (*k* = 9000), retaining a regular polygon shape in the simulation, assisted by a harmonic potential with the same strength applied to each angle. For each polygon at a given pressure, a regular MD simulation was performed for 1.2 × 10^7^ steps starting from the ground‐state crystalline morphology at a temperature that is 0.01 lower than the transition point, and then the last snapshot of the simulation is used as the initial configuration for the heating process. The method used to determine the phase transition point has been described in ref. [48] and the related data can be found in the corresponding data repository.^[^ ^67^ ^]^

Due to the hysteresis of first‐order phase transition, the transition point determined by heating is higher than the one determined by cooling. Therefore, two kinds of initial configurations were prepared for the reverse process (cooling process). The first kind of initial configurations are prepared by equilibrating the rotator crystal phase obtained from the s–s transition described above, and the second ones are prepared by cooling down the rotator crystal phase to a temperature 0.01 higher than the phase transition point of the reverse process, and then relaxed for 1.2 × 10^7^ steps. Our simulations indicate that the results of the reverse process do not depend on initial configurations.

### MD Simulations

All the simulations in this work were conducted with LAMMPS.^[^ ^68^ ^]^ Starting from the equilibrated structures of the parent phase, the simulations were performed in the isothermal‐isotension ensemble^[^ ^69^ ^]^ at *T*_m_ and *T*_m_ + 0.01 with the periodic boundary conditions applied to both *x* and *y* directions, where *T*_m_ is the phase transition point. The s–s transition process can be observed within 1.6 × 10^7^ steps, evidenced by the increase of potential energy and the decrease of density. To determine the phase transition rate, ten independent simulations were further performed initiating from four different configurations (the last or the penultimate snapshots of the simulations at a temperature that is 0.01 or 0.02 lower than the phase transition point), and evaluated the standard error under each thermodynamic condition. Five of these ten simulation trajectories are simulated at *T*_m_ and the other half are simulated at *T*_m_ + 0.01. The results are found to be insensitive to the small degree of superheat and the initial configuration. Furthermore, simulations were also performed at *T*_m_ + 0.1 at the lowest pressure for each polygon. The patterns of the body‐orientational field remain unchanged, as shown in Figure S16 (Supporting Information), while the phase transition is significantly accelerated, manifesting our conclusions are robust with respect to a wide range of temperatures. For the reverse process, the initial configurations are simulated for 1.6 × 10^7^ steps at a temperature that is 0.01 lower than the corresponding phase transition point to observe the s–s transition. Once the reverse trajectory ends in polycrystalline morphology, it never equilibrates into a perfect crystal even with an extended simulation time up to 3.2 × 10^7^ steps.

L‐J units are used in this work, and set L‐J parameters *m* = 1, $\sigma =\sqrt[{6}]{2}\;$, and ε  =  5. The cutoff radius of the L‐J interaction is set to be *r_c_* =  7, ensuring a high accuracy on the estimation of the potential energy. The pressure and temperature are controlled by the Nóse‐Hoover barostat and thermostat,^[^ ^70^, ^71^, ^72^, ^73^ ^]^ respectively. The time integration is performed by the velocity‐Verlet algorithm with a timestep of 0.005. The strict 2D nature is realized by performing time integration on *x* and *y* directions and setting all *z* coordinates as 0. A more concrete discussion on the selection of the parameters can be found in our previous work.^[^ ^48^ ^]^

For the heating process, the s–s transition was investigated at various pressures to ensure our conclusions are robust. The lowest pressure that is studied is the lowest one at which the rotator crystal can stabilize, i.e., *P* = 0 for pentagon and octagon while *P =* 1.5 for hexagon. The highest pressure that is examined is *P* = 10 for pentagon, *P* = 7.5 for hexagon, and *P* = 8 for octagon. A higher pressure for octagon can easily cause numerical instability in MD simulations as reported in the previous study,^[^ ^48^ ^]^ and a higher one for hexagon leads to numerical instability in fixed simulations. Only the case at *P* = 0 has been studied for the reverse process, since a relatively high pressure may easily cause numerical instability in the simulations.

In the fixed simulation, each monomer is treated as a rigid body, whose translational motion is eliminated in each step to ensure a fixed COM. The temperature is controlled by the Langevin dynamics. The timestep in the fixed simulations is reduced to 0.001 to avoid numerical instability. Compared to the original ball‐stick polygon, the motions of all the hard modes, i.e., the vibration of the bonds and the angles, are removed for a rigid body, but it can be proved that this has no influences on the thermodynamic free energy landscape.^[^ ^74^ ^]^ Eight points were selected out from each unfixed MD trajectory to serve as the starting points to run 8 individual fixed simulations. The error‐bars of the data points in Figure 2b and Figures S12 and S14 (Supporting Information) represent standard deviations, resulting from the thermal fluctuations in one fixed simulation trajectory.

### Data Analysis with Body‐Orientation Field

The angle of each unit vector is given by the argument of the body‐orientational order parameter with respect to the *x*‐axis. The local defect is defined by $\oint \nabla \left(n\theta_{i}\right)\cdot d\;\vec{S}=2\pi q_{i}$, which has the same expression as for the topological defect in 2D systems except that the integration here is taken on the smallest closed loop around each lattice point rather than globally. First provided for the studies of the XY model and 2D crystals,^[^ ^75^ ^]^ this quantity has now been applied to a variety of systems including liquid crystal,^[^ ^76^ ^]^ cell tissue,^[^ ^64^ ^]^ and amorphous solid,^[^ ^77^, ^78^ ^]^ concentrating on diverse vector fields. As shown in Figures S3–S11 (Supporting Information), the locations of the defects highly coincide with the places where body‐orientations change drastically, manifesting that the local defects capture the essential features of the amorphization process in the body‐orientation field. This can also be evidenced by the synchronous time evolution of the defect concentration and the body‐orientational order parameter as shown in Figure S2 (Supporting Information). Moreover, it is obvious that the positive charges are much more than the negative ones, different from the case for topological defects, where the system must stay neutral to ensure a finite energy,^[^ ^75^ ^]^ demonstrating that the local defects that are defined here are not topological ones.

The spatial distribution of the defects during the phase transition process is quantified by dividing the simulation box into 32 × 31 equal cells and then averaging the number of defects in all sampled configurations along the transition. We have also double‐checked by dividing the box into 26 × 24 cells and found that the patterns of local defects are qualitatively the same as those shown in Figure 2. To further ensure the robustness of these patterns, the same analysis was performed for another three sets of individual trajectories under the same conditions at the lowest pressures, and found no qualitative differences.

### Finite Size Effects

All the results reported in the main text were obtained from the simulations with 2496 (52×48) monomers. larger simulations with 4620 (70×66) monomers were further performed to estimate the finite‐size effect. As shown in Figure S17 (Supporting Information), the pattern for each polygon is qualitatively the same. Specifically, the striped region for pentagon is clearer in the larger system, which further verifies the vague striped region in the body‐orientation field shown in Figure 1d. Moreover, there can be two striped regions in the larger pentagon system, since a larger system can provide more “nucleation sites.” More discussions can be found in Supporting Information.

## Conflict of Interest

The authors declare no conflict of interest.

## Author Contributions

R.Z. conceived and designed the research. R.Z. performed the simulation and analyzed the data under the supervision of Y.W. All the authors discussed the results and wrote the paper.

## Supporting information



Supporting Information

## Data Availability

The data that support the findings of this study are available from the corresponding author upon request.
