# Supplementary material for: Shape‐Determined Kinetic Pathways in 2D Solid–Solid Phase Transitions
Source: Adv Sci (Weinh). 2025 Nov 3;13(4):e17016. doi: 10.1002/advs.202517016 (PMC12822416; doi:10.1002/advs.202517016)
Supplement: Supplementary file 1 — Supporting Information [file ADVS-13-e17016-s001.pdf]

Supplementary Information for: Shape-Determined Kinetic Pathways in 2D Solid-Solid Phase Transitions

Ruijian Zhu[1,3], Yi Peng[2,3], Yanting Wang[1,3] .

[1] Institute of Theoretical Physics, Chinese Academy of Sciences, Beijing 100190, China
[2] Beijing National Laboratory for Condensed Matter Physics, Institute of Physics, Chinese Academy of Sciences, Beijing 100190, China
[3] School of Physical Sciences, University of Chinese Academy of Sciences, 19A Yuquan Road, Beijing 100049, China

This PDF file includes:

Supplementary Text
Figure. S1 to S17
Table S1
Reference 1

S1. Robustness of Body-Orientation Fields at Various Pressures

In Table S1, we list the differences of thermal quantities under various pressures, manifesting that the structures of two solid states are more similar at a higher pressure. This demonstrates that the energy barrier for rotational motion decreases as pressure increases. Moreover, the body-orientational order parameter for pentagon takes a value about 0.7 in the parent phase, much less than about 0.85 for hexagon and octagon, indicating that local defects are easier to be created even before transition, as evidenced in Figure S3(a). It is worth mentioning that the s-s transition of pentagon and octagon can occur at an arbitrary pressure including P = 0, but the one of hexagon occurs only at $P \geq 1.5$. This is because the close-packing phase of hexagon is ultra-stable at low pressures, as a consequence of the perfect match of interaction and shape, allowing it stabilizes up to a relatively high temperature, and then melts into fluid state directly[1].

In Figure S3-S11, we show the time evolution of the body-orientation field for each polygon at various pressures. In each figure, the pointing direction of each arrow represents the argument of the body-orientation order parameter ϕ with respect to the x-axis, while the purple and blue dots label the positions of local defects with positive and negative charges, respectively. It is evident that the majority of the dots in these figures are colored in purple, suggesting that the system has a net positive 'charge'. This implies that the local defects we define here are not topological defects, but rather indicators of the regions where body-orientations change dramatically with respect to neighboring monomers.

The time evolution of the local defects described in the main text is robust at different pressures, as demonstrated by performing statistical analysis on the spatial distribution of local defects. The results are depicted in Figure S12, where pentagon exhibits one or two vague stripe regions with several isolated warm-colored points, hexagon shows no collective patterns, and octagon consistently displays a distinct stripe region. The fact that pentagon can have more than one stripe region should be attributed to the lower energy barrier at higher pressures, which also slightly fuzzes up the stripes.

In Figure S3-S11, we also label the corresponding MD steps for each panel. Comparing the time intervals between different panels in Figure S5, Figure S8, and Figure S11, which present the evolution of the body-orientation fields for octagon at different pressures, one can identify that the time required for forming a narrow stripe region across the box is very short and quite stable at different pressures, but further growth along the normal direction is significantly accelerated at higher pressures, consistent with the fact that the energy barrier for rotational motion is lower at higher pressures. Recalling that the thermodynamic quantities of pentagon have the same tendency as those of octagon with increasing pressure, it is expected that the growth rate for pentagon should follow the same trend as octagon. This can indeed be observed as in Figure S3, S6, and S9, despite the smearing of the stripe regions. As shown in Figure S4, S7, and S10, the behavior of hexagon is entirely different: The transition process is dominated by translational motion and no stripe regions appear due to its weak dependence on the energy barrier, leading to an almost constant transition rate invariant to pressure.

S2. Details of Fixed Simulations and Robustness of Kinetic Pathways

In the fixed simulations for each polygon at each pressure, the initial configurations are selected along the original unfixed MD trajectory with the occurrence of the s-s transition.

This is illustrated at the lowest pressures for different polygons in Figure S13(a)-(c), in which each initial configuration for the corresponding fixed simulation is marked as a colored point along the time-evolution curve for the body-orientational order parameter in the unfixed (original) MD trajectory. The time evolutions of the body-orientational order parameter in the fixed simulations at the lowest pressures for different polygons are shown in Figure S13(d)-(f). The same procedure is employed for the fixed simulations at other pressures, with the time evolutions of the body-orientational order parameter being qualitatively the same. In Figure S14, we plot the results from the fixed simulations at various pressures for each polygon, demonstrating the robustness of the kinetic pathways with respect to thermal conditions. There are fewer available data points at higher pressures because numerical instability causes the fixed simulations to have a strong tendency of breaking down. The fixed simulations for the reverse processes follow the same procedure, whose results are shown in Figure S15. It can be seen that a polygon system may have more than one kinetic pathway for the reverse process. However, it is evident that a kinetic pathway dominated by translational motion at the initial stage consistently traps into a polycrystalline morphology, and always ends up with a perfect crystal state otherwise. All the average values for the fixed simulations shown here and in the main text are calculated after the constrained systems are equilibrated.

S3. Finite-Size Effects

To eliminate possible finite-size effects, we further performed simulations on the systems composed of 4620 polygons at $P = 0$ for pentagon and octagon as well as at $P = 1.5$ for hexagon. These simulations follow the same procedure as the ones for 2496 polygons. As shown in Figure S17, the patterns of local defects in the body-orientation field for different polygons are qualitatively the same. Specifically, pentagon can exhibit one or two crossed striped regions, as depicted in Figure S17(a) and (b), since a larger system provides more ‘nucleation sites’, enabling the spontaneous formation of two striped regions, benefiting from the relatively easy rotational motion. Furthermore, the striped regions in these two panels are clearer than the ones in Figure 2(a), Figure S12(a), and Figure S12(d), manifesting that the formation of striped region persists in larger systems.

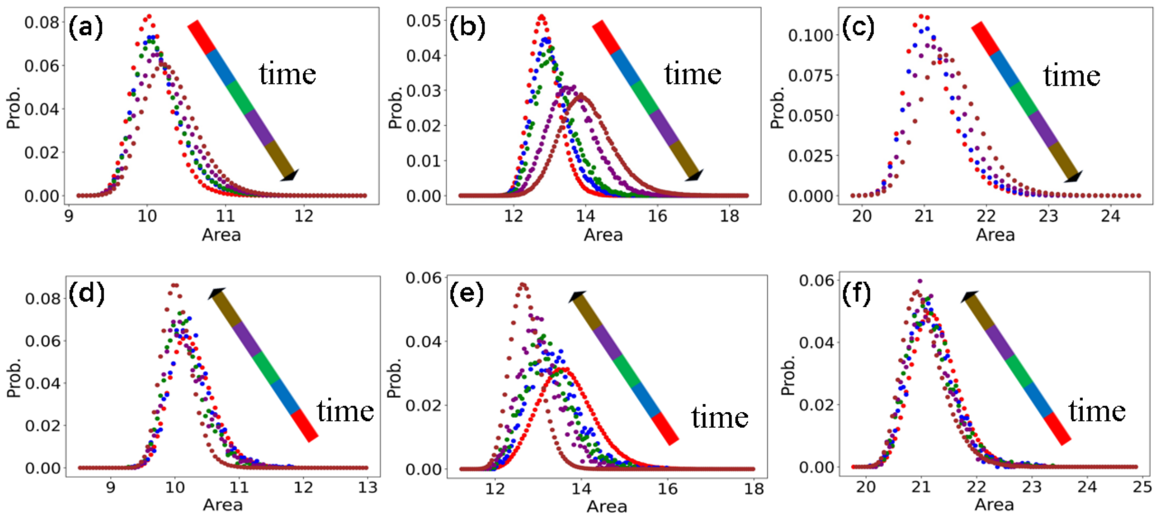

Figure S1. Time evolutions of the distributions of Voronoi cell area in the heating (a)-(c) and cooling (d)-(f) processes of s-s transition for pentagon, hexagon, and octagon. (a) and (d) for pentagon, (b) and (e) for hexagon, (c) and (f) for octagon. The probability density (Prob.) always exhibits a single-peak distribution, while the peak value moves continuously towards the direction corresponding to a larger (for heating process) /smaller (for cooling process) cell area, which is in consistent with the theoretical prediction of homogeneous expansion/contraction for isostructural s-s transition.

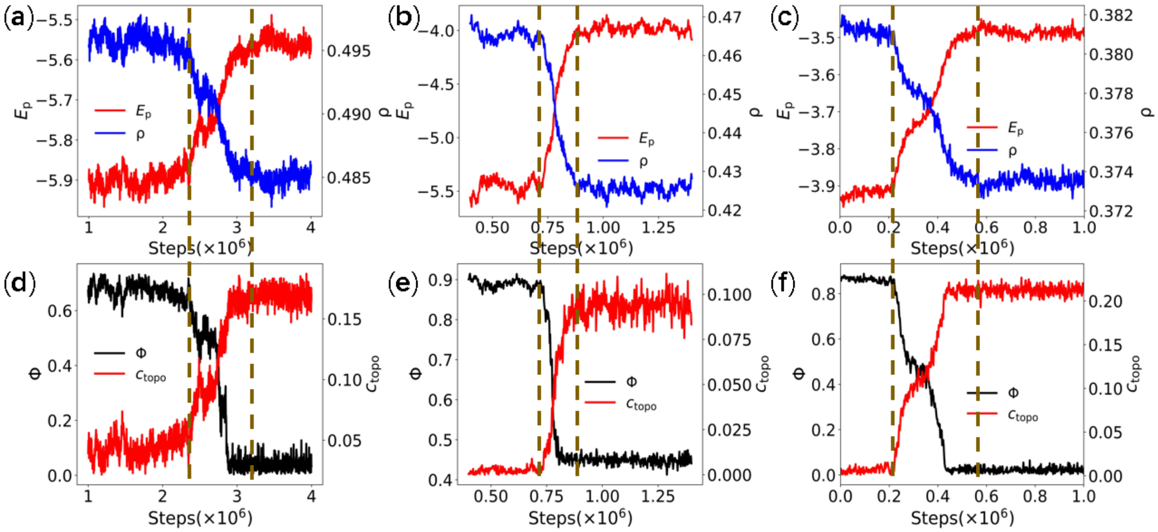

Figure S2. Time evolutions of the collective quantities during s-s transition. (a)-(c) for the evolution of potential energy and density, (d)-(f) for the evolution of body-orientational order parameter and concentration of local defects. (a) and (d) for pentagon, (b) and (e) for hexagon, (c) and (f) for octagon. The dashed brown lines are used to guide the eyes for the initiating and ending points of the transition. It can be seen that different quantities start to rise up at almost the same time, while the potential energy and density take several more steps to approach their final values than the body-orientational order parameter and concentration of local defects. It is also evidenced that the concentration of local defects varies synchronously with the body-orientational order parameter.

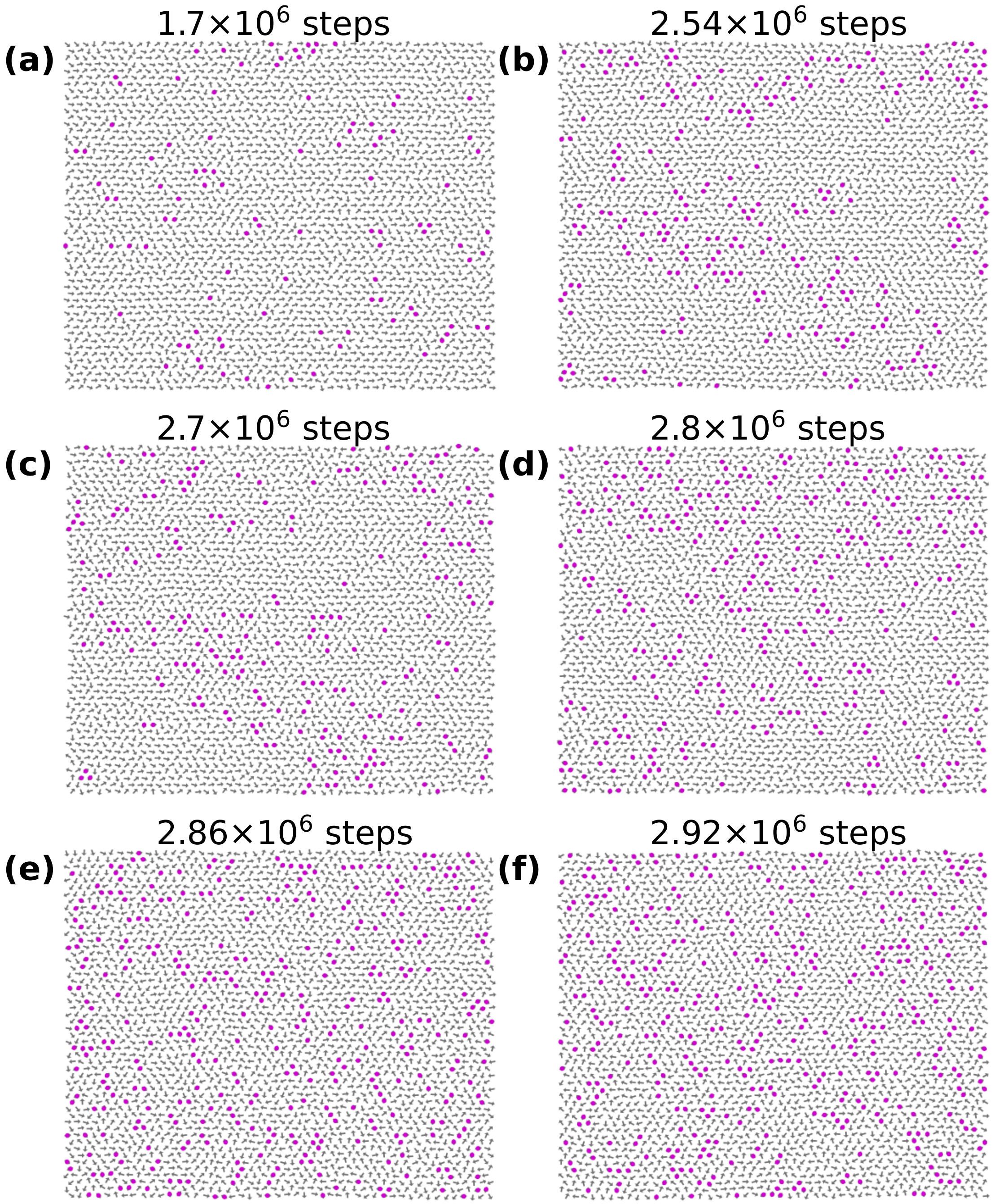

Figure S3. Defects of pentagon at *P* = 0. Each arrow located on the COM of a ball-stick polygon represents the argument of the corresponding body-orientational order parameter with respect to the *x*-axis. The dots mark the positions of the local defects in the body-orientational field, colored in purple and blue (can hardly be seen here) dots for positive and negative 'charges', respectively. The same plotting scheme is also applied to Fig. S2-S9. The parent phase shown in (a) already contains several local defects. After forming a vague stripe region shown in (b), the number of defects increases with time, as shown in (c)-(e). Finally, as shown in (f), the system transforms into a rotator crystal with a large number of uniformly distributed local defects.

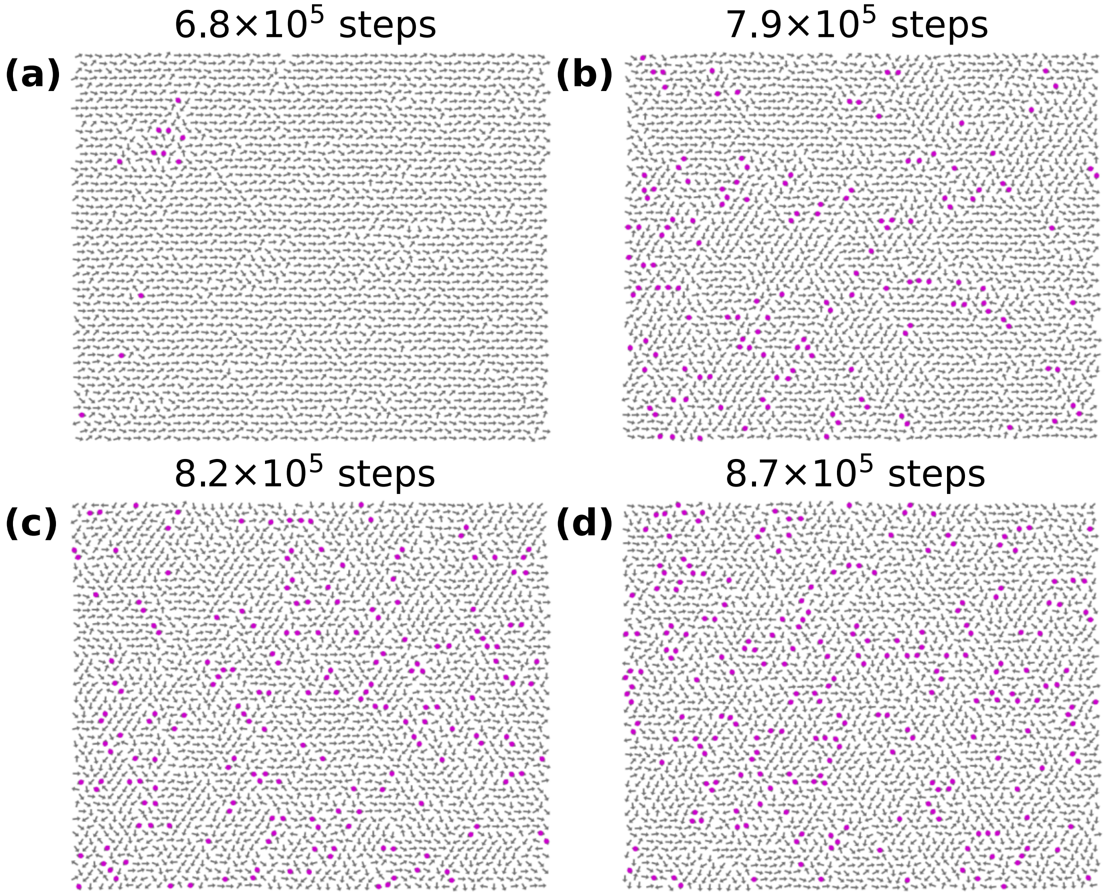

Figure S4. Defects of hexagon at *P* = 1.5. There are very few defects at the initial stage shown in (a). The number of defects then increases with time during the transition process, as shown in (b)-(d), with no evidence of a collective pattern.

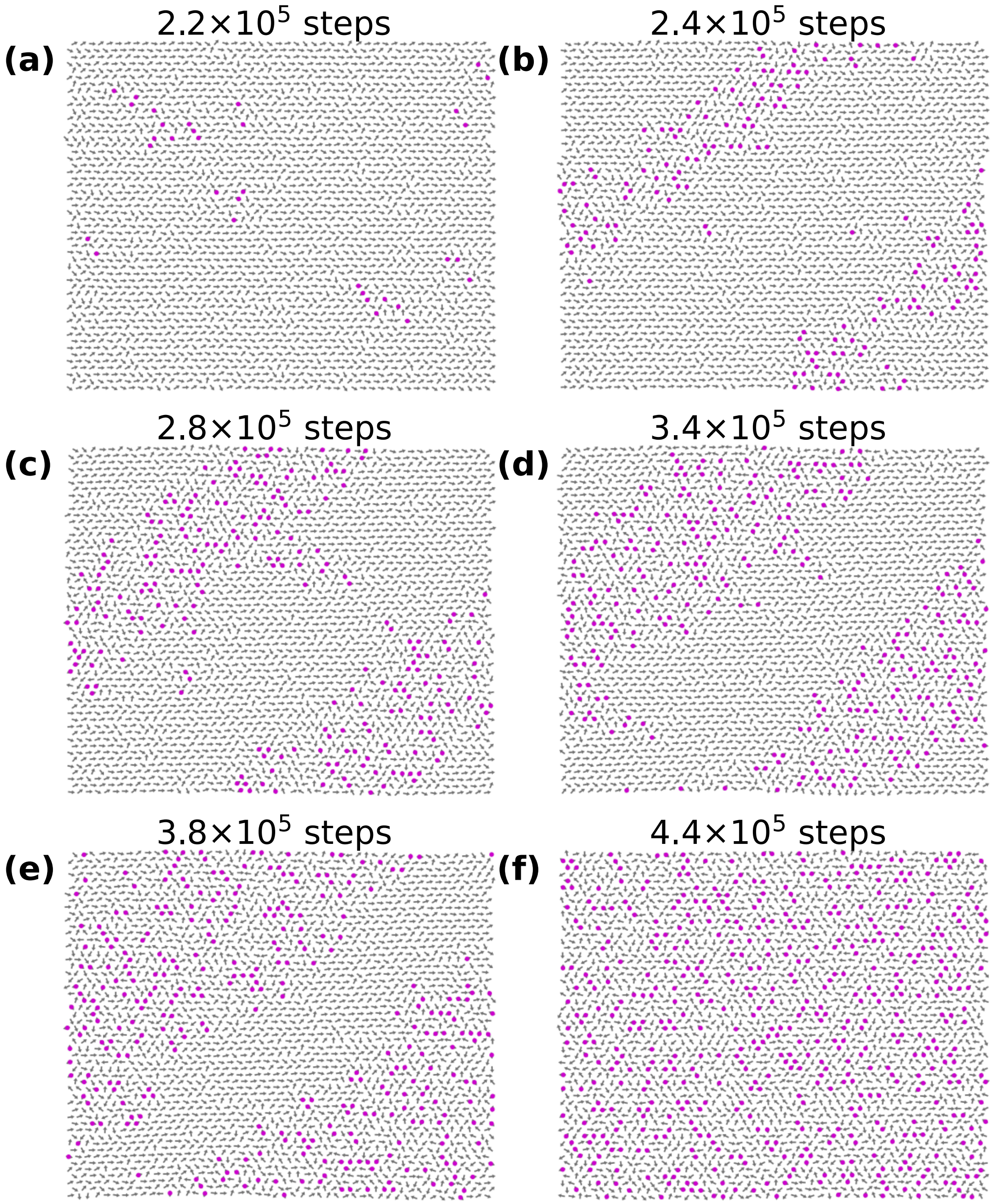

Figure S5. Defects of octagon at *P* = 0. Similar to hexagon, only a few defects present at the initial stage, as shown in (a). As shown in (b), after a very short time, the defects form a narrow stripe region across the periodic simulation box. Then the stripe region attempts to grow along its normal direction, as shown in (c)-(e). Finally, as shown in (f), when the stripe uniformly covers the whole simulation box, the system transforms into the rotator crystal phase.

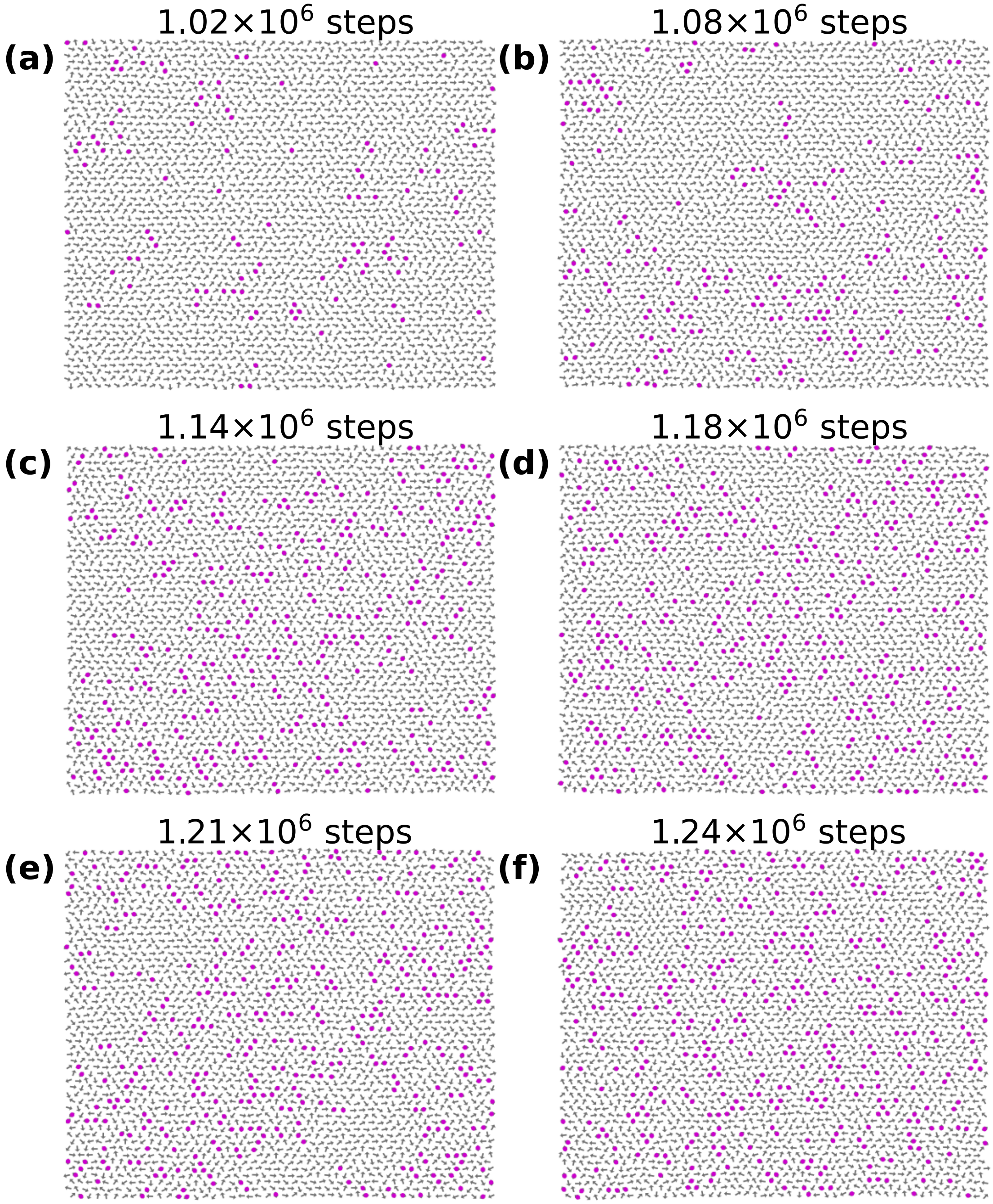

Figure S6. Defects of pentagon at *P* = 5. It is similar to the case at *P* = 0, but the growth after forming a vague stripe region is significantly accelerated.

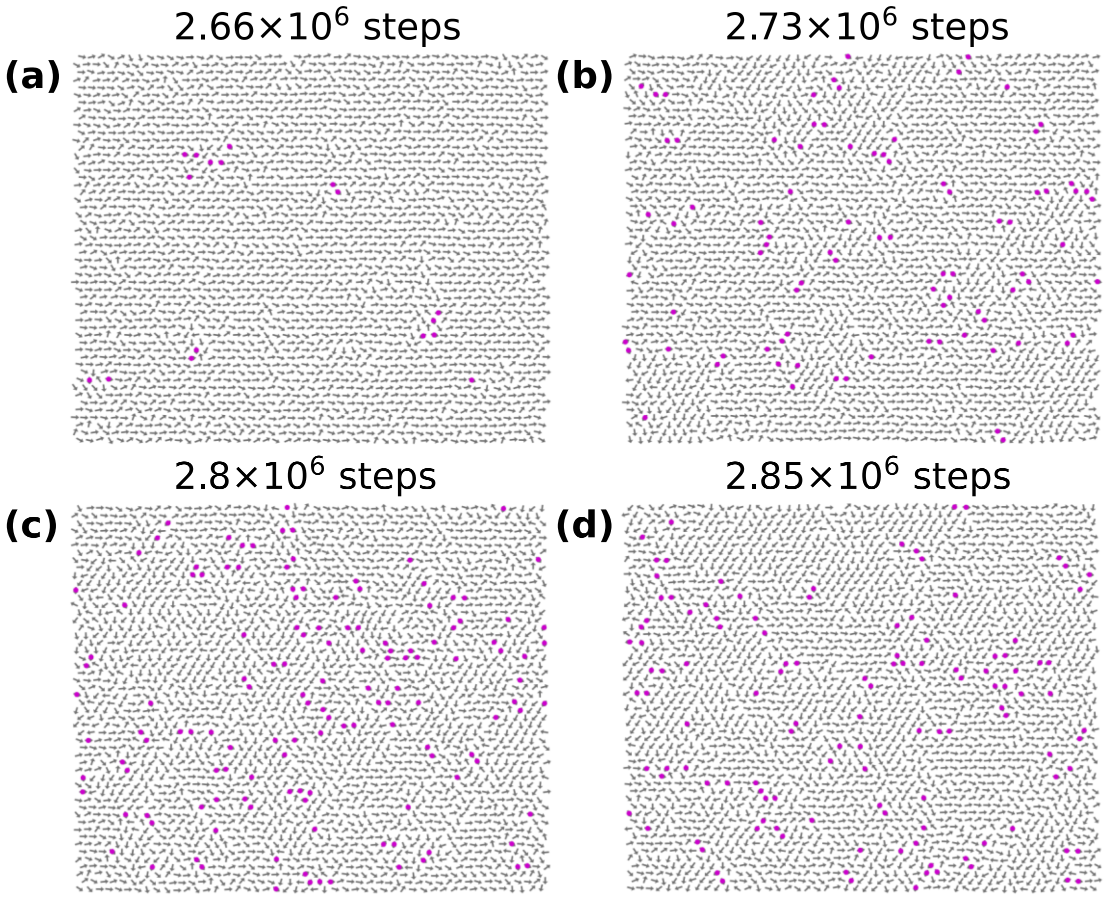

Figure S7. Defects of hexagon at *P* = 5. It is similar to the case at *P* = 0.

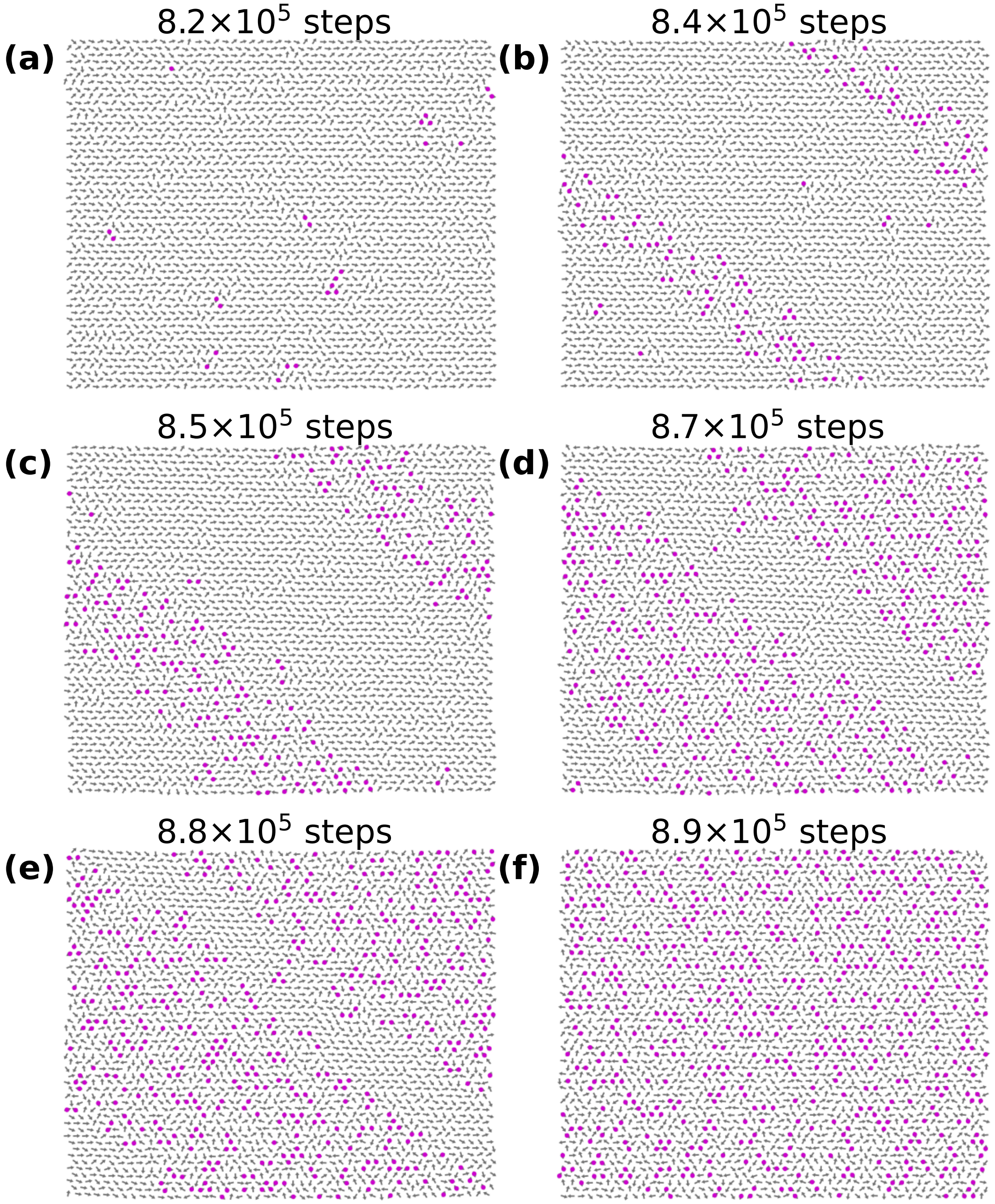

Figure S8. Defects of octagon at *P* = 5. It is similar to the case at *P* = 0, but the growth of the stripe region along its normal direction is significantly accelerated.

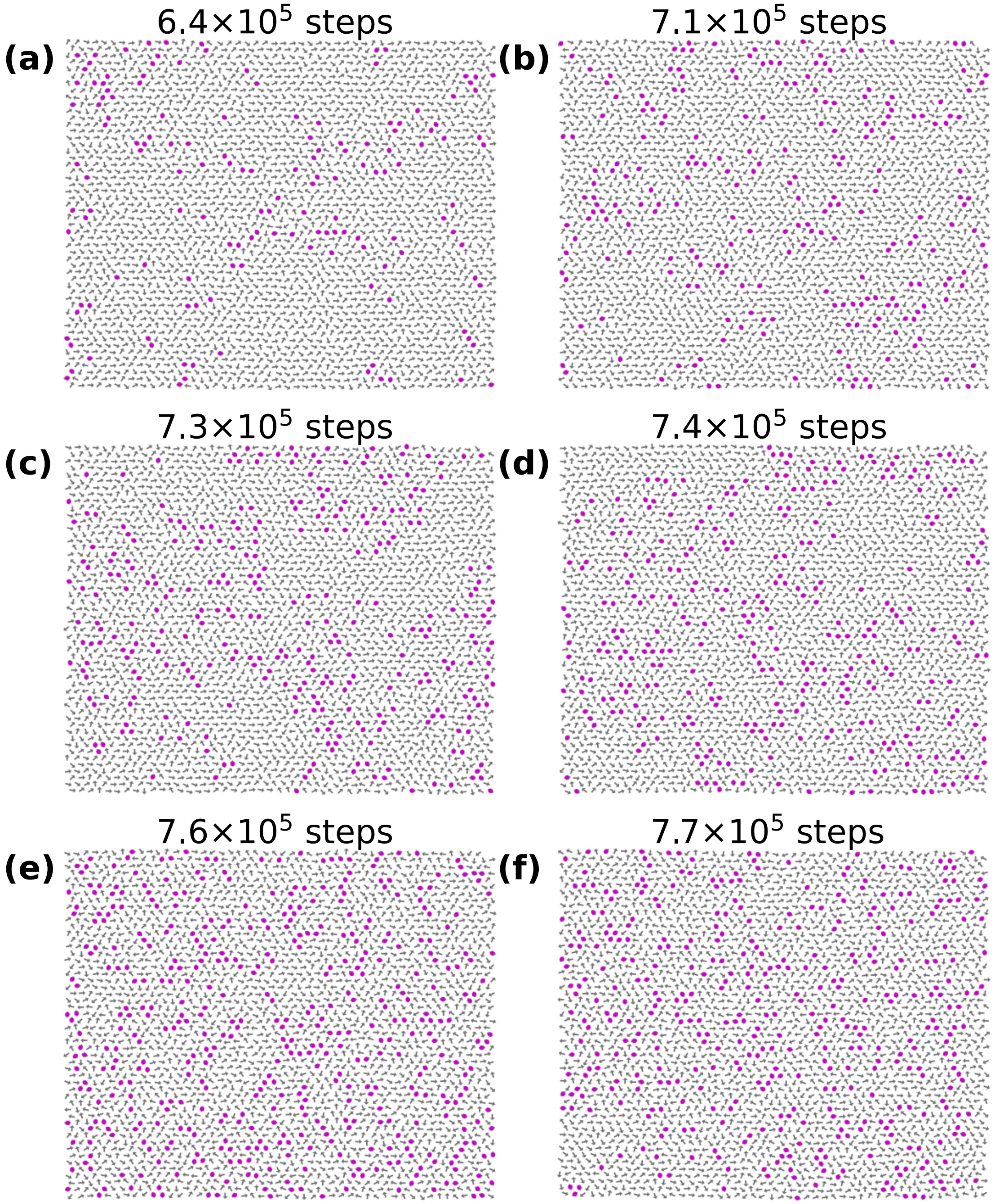

Figure S9. Defects of pentagon at *P* = 10. It is similar to the case at lower pressures, but the growth after forming a vague stripe region is further accelerated.

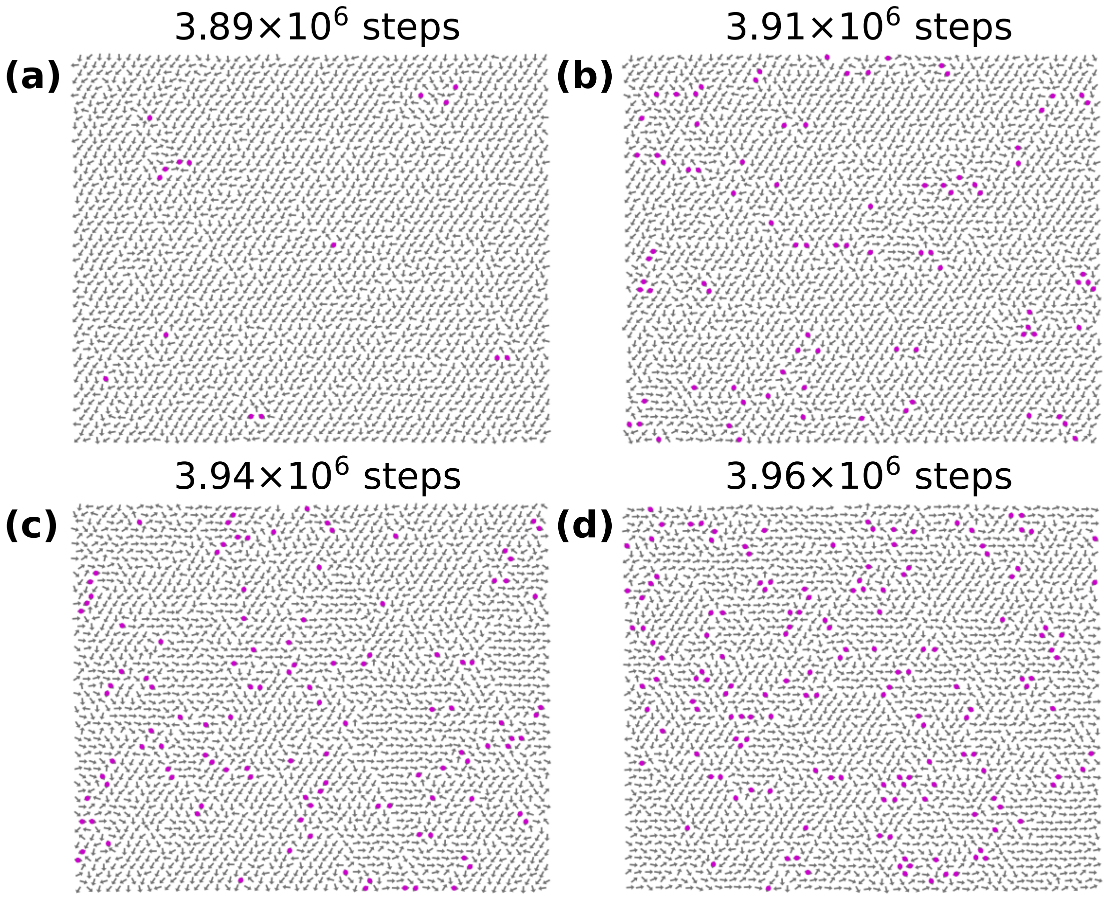

Figure S10. Defects of hexagon at *P* = 7.5. It is similar to the case at lower pressures.

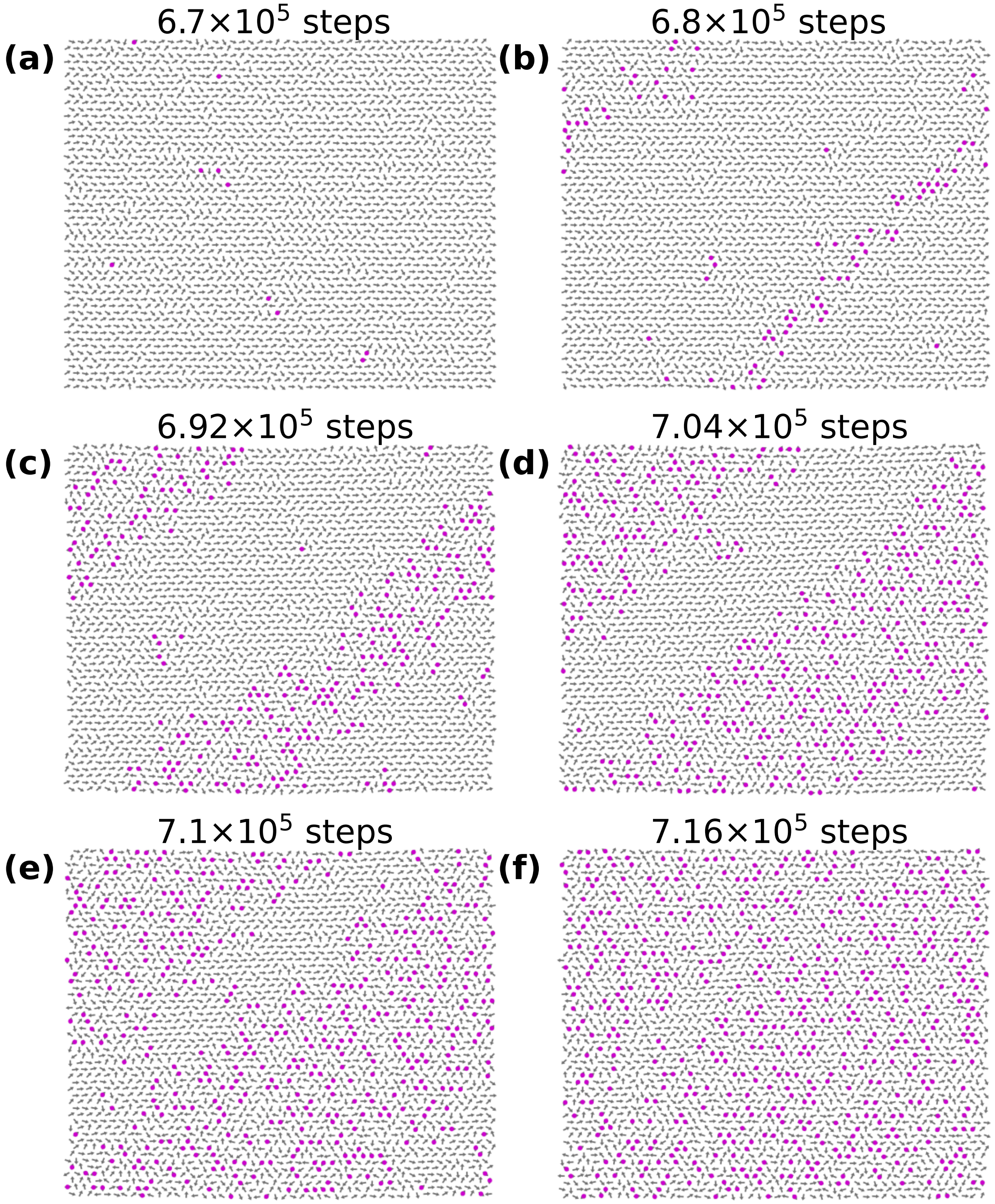

Figure S11. Defects of octagon at *P* = 8. It is similar to the case at lower pressures, but the growth after forming a vague stripe region is further accelerated.

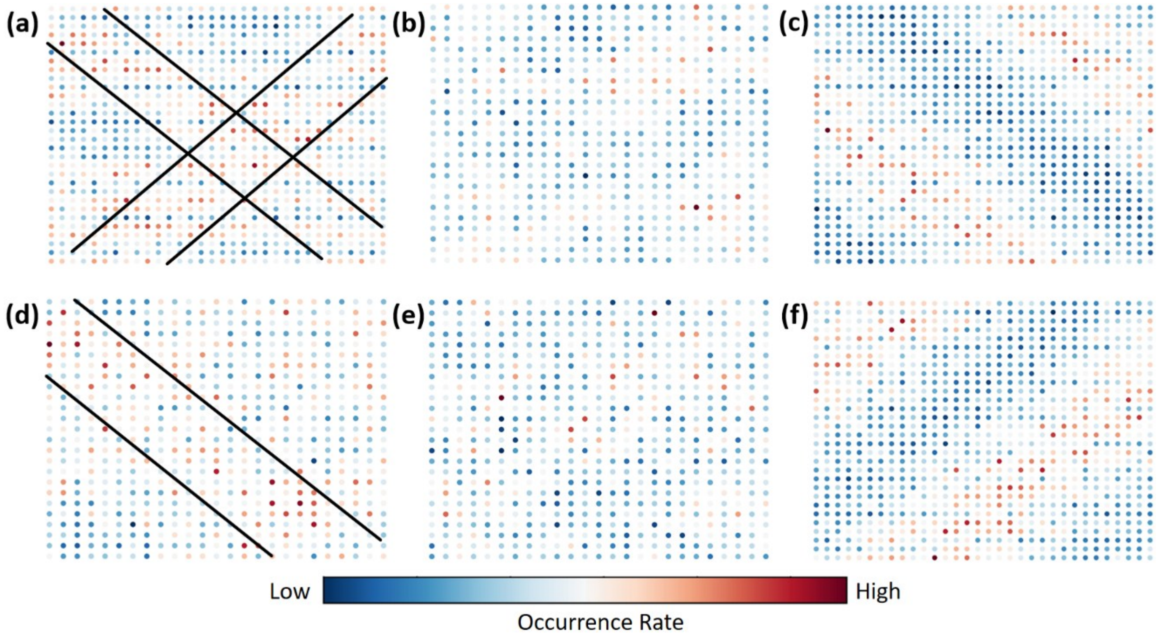

Figure S12. Statistics of local defects at various pressures. (a) Pentagon at P = 5. (b) Hexagon at P = 5. (c) Octagon at P = 5. (d) Pentagon at P = 10. (e) Hexagon at P = 7.5. (f) Octagon at P = 8. In (a) and (d), the black lines are drawn to highlight the vague stripe regions.

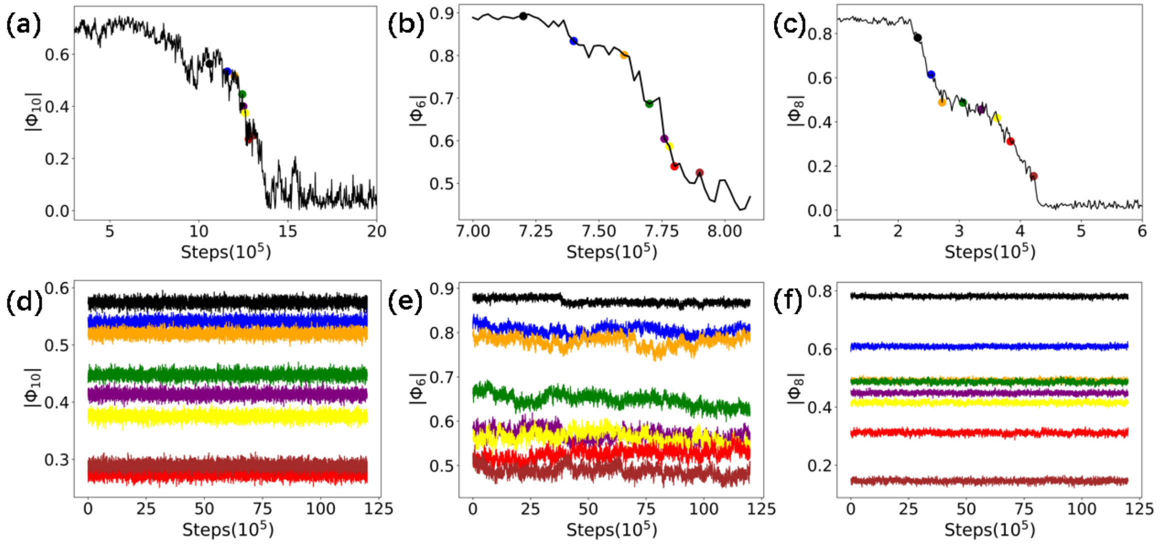

Figure S13. Selection of initial configurations and simulation results of the fixed simulations at the lowest pressures. In (a) - (c), the black line is the time evolution of the body-orientational order parameter, and the colored points mark the moments when the initial configurations for the fixed simulations are selected. In (d) - (f), we show the time evolutions of the body-orientational order parameter in the 'fixed' simulations, (a) and (d) for pentagon, (b) and (e) for hexagon, and (c) and (f) for octagon. The coloring of the lines in (d) - (f) is the same as the points in (a) - (c).

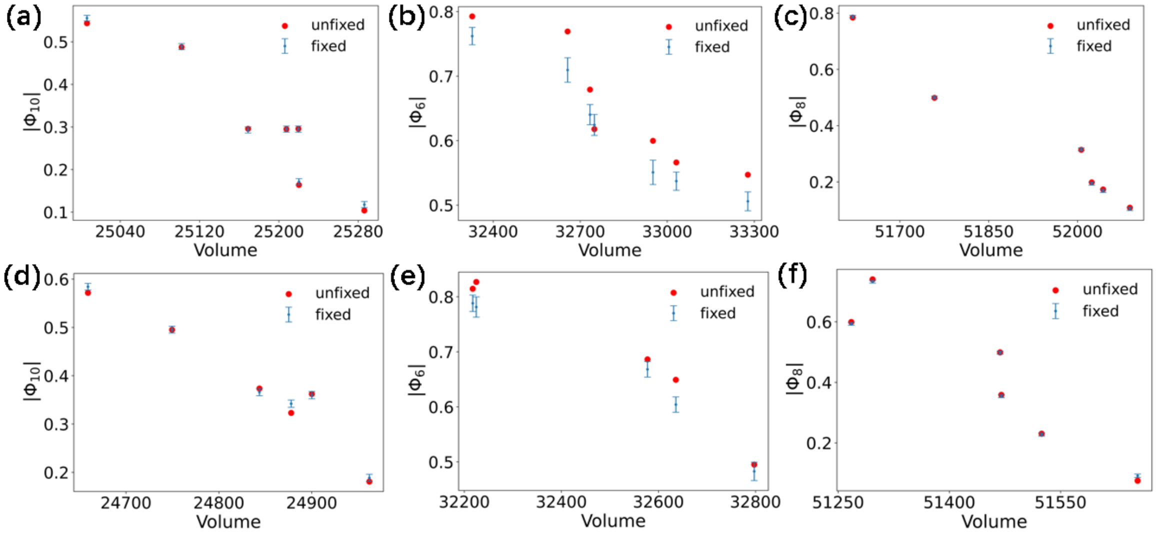

Figure S14. Results from the fixed simulations at various pressures for the heating process. (a) Pentagon a P = 5. (b) Hexagon at P = 5. (c) Octagon at P = 5. (d) Pentagon at P = 10. (e) Hexagon at P = 7.5. (f) Octagon at P = 8. Compared with the ones shown in Figure 2 in the main text, it can be found that the kinetic pathway of a specific polygon is independent of thermal conditions.

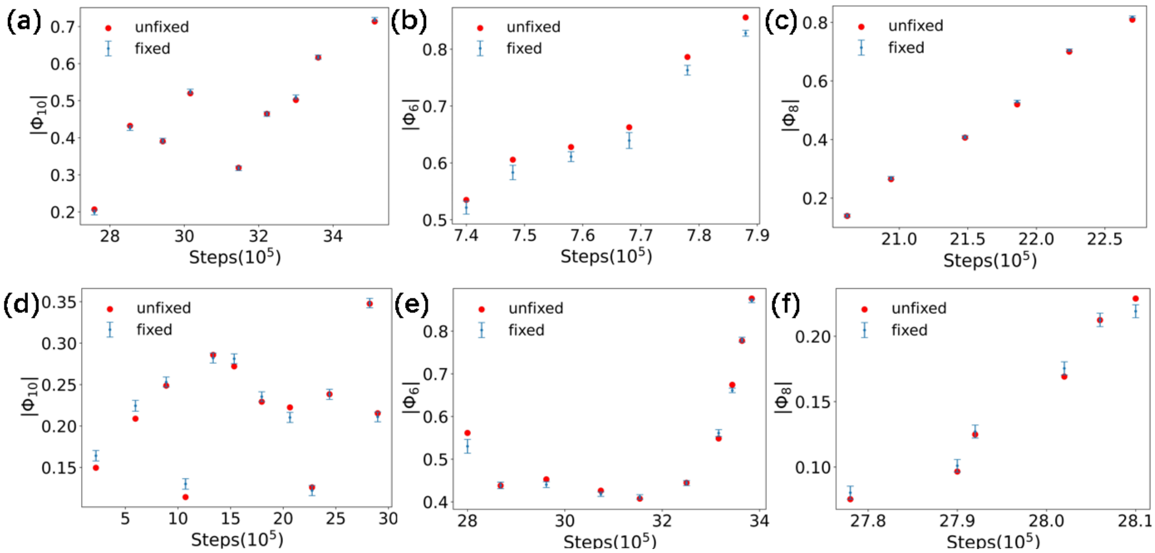

Figure S15. Body-orientational order parameters in the fixed simulations for the reverse process. The 'steps' are those in the unfixed MD simulation. Since some of the trajectories are trapped into the polycrystalline state, only the comparison at the early stage is meaningful, which determines whether the system can modify its body-orientational order before contracting into a dense state. (a) and (d) for pentagon, (b) and (e) for hexagon, (c) and (f) for octagon.

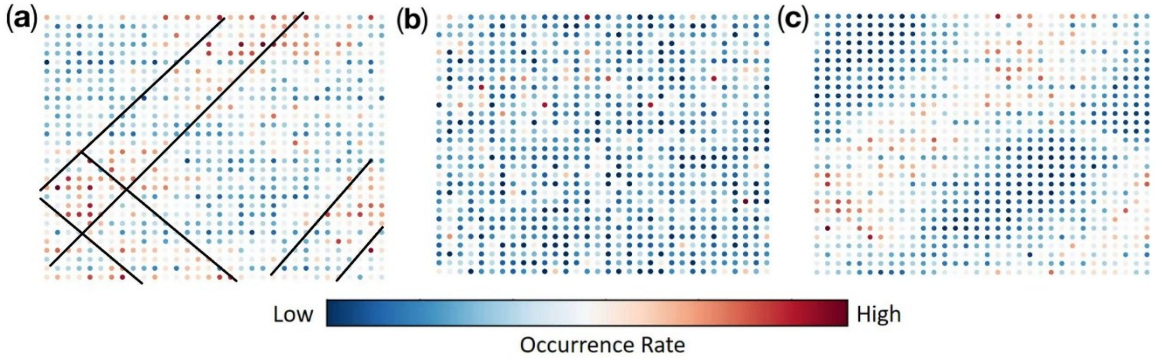

Figure S16. Statistics of local defects in the body-orientation fields at the temperature of T_m + 0.1. (a) Pentagon, (b) Hexagon and (c) Octagon. The black lines are used to guide the eyes for the stripes. It can be found that the patterns are more smeared but qualitatively the same as the cases at a lower degree of superheat.

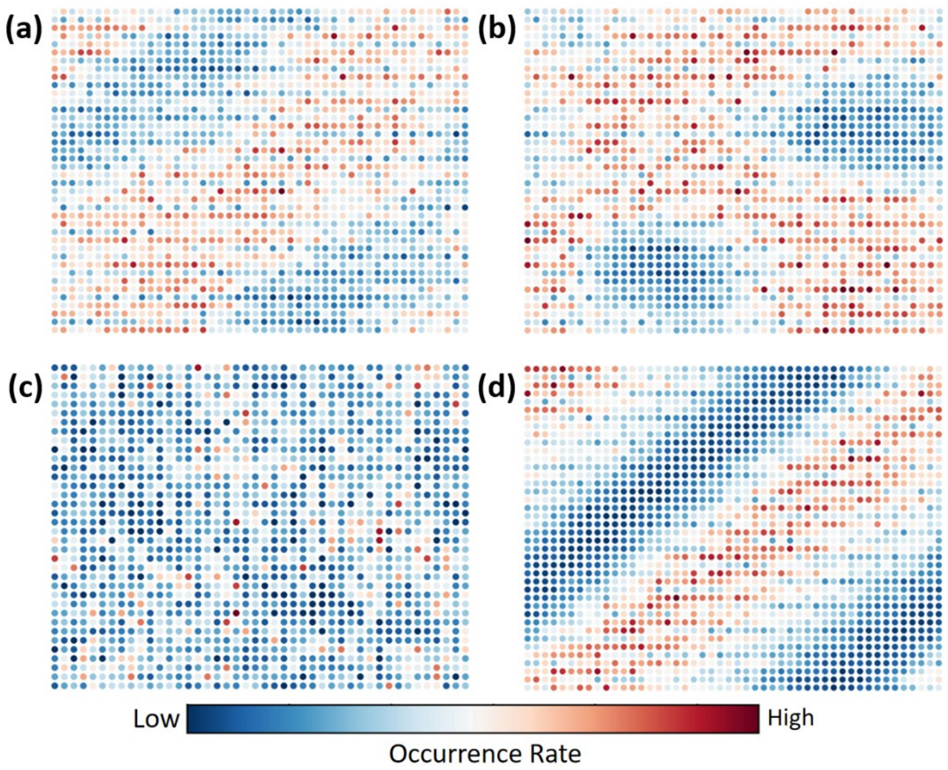

Figure S17. Statistics of local defects in the body-orientation fields for *N* = 4620. (a) and (b) are for pentagon, indicating that it is possible to form one or two stripe regions in a larger system. (c) for hexagon and (d) for octagon.

Table S1. Differences of thermodynamic quantities between the parent phase and the product phase at the melting points under various pressures.

Shape	Pressure	ΔE_{p}	$\Delta \rho^{-1}$	$\Delta \Phi$
**Pentagon**	$P = 0$	0.38531	0.049953299	0.71041631
	$P = 5$	0.31036	0.038348263	0.66040313
	$P = 10$	0.29444	0.035007953	0.65213229
**Hexagon**	$P = 1.5$	1.449	0.195133792	0.443682344
	$P = 5$	0.77023	0.090753697	0.415460196
	$P = 7.5$	0.57863	0.066392551	0.395135211
**Octagon**	$P = 0$	0.42008	0.052412336	0.851954527
	$P = 5$	0.35332	0.032927826	0.8297257
	$P = 8$	0.34004	0.028727551	0.822012797

Reference

1 R. Zhu and Y. Wang, A critical edge number revealed for phase stabilities of two-dimensional ball-stick polygons. *Nat. Commun.* **15**, 6389 (2024).
